# Recent progress in the interaction between energetic particles and tearing modes

**DOI:** 10.1093/nsr/nwac019

**Published:** 2022-02-15

**Authors:** Huishan Cai, Ding Li

**Affiliations:** Chinese Academy of Sciences Key Laboratory of Geospace Environment, School of Nuclear Sciences and Technology, University of Science and Technology of China, Hefei 230026, China; Institute of Physics, Chinese Academy of Sciences, Beijing 100190, China; Songshan Lake Materials Laboratory, Dongguan 523808, China

**Keywords:** magnetically confined plasma, energetic particle, tearing mode, transport, confinement, tokamak

## Abstract

The dynamics of energetic particles and tearing modes and the interactions between them are of great significance for magnetically confined fusion plasmas. In this review, we focus on these issues in the context of tokamak plasmas. The interaction between energetic particles and tearing modes is considered from two perspectives: (i) the influence of energetic particles on tearing modes and (ii) the transport of energetic particles by tearing modes. The influence of energetic particles on tearing modes is described on the basis of a general dispersion relation for tearing modes. The effects of energetic particles are considered separately in the outer region and the island region of a tearing mode. The physics mainly results from the modification of the perturbed parallel current by energetic particles without wave–particle resonance. In addition, the resonance between energetic particles and tearing modes is also reviewed. For the transport of energetic particles, transport of both circulating and trapped energetic particles by tearing mode is reviewed. Our descriptions of physical phenomena here are based on an analytical approach, while the experiments and simulations are used to illustrate and confirm our results. Finally, a number of open issues are discussed.

## Teaser

One of the critical physics problems in magnetically confined fusion plasma, that the interaction between energetic particles and tearing modes, is reviewed based on theory, simulation and experiment.

## INTRODUCTION

**Figure 1.**
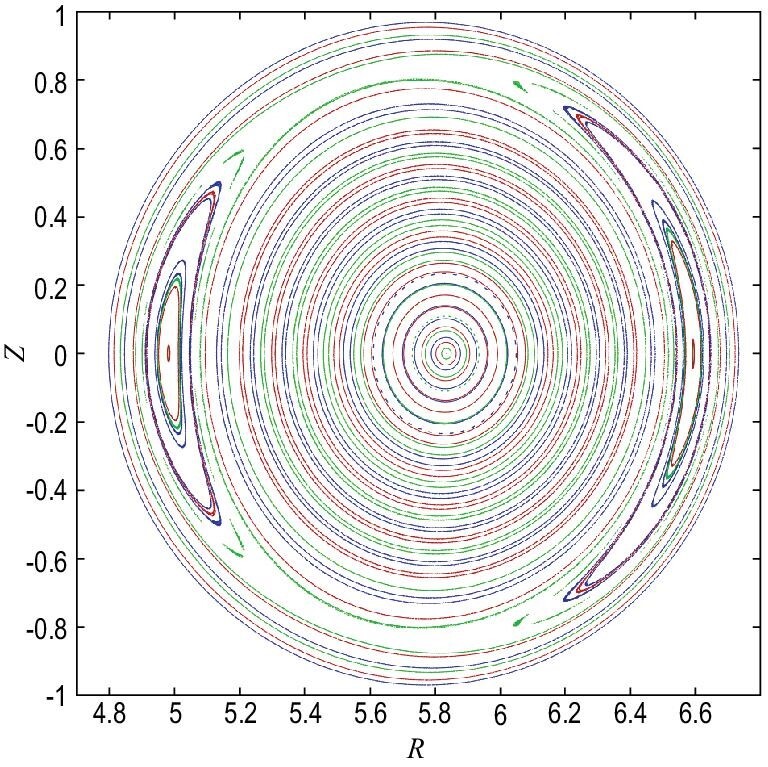
Magnetic island at the safety factor *q* = 2/1 surface.

Tearing modes (TMs) are among the most dangerous instabilities in a magnetically confined plasma. They are driven by the radial gradient of the equilibrium toroidal current density [1], with the topology of the magnetic field being changed to form a magnetic island (MI) owing to the finite resistivity (shown in Fig. 1), which can increase the local transport and degrade plasma confinement. In a fusion plasma, the occurrence of TMs (including neoclassical TMs (NTMs)) is an important issue. In future magnetically confined fusion devices, such as the International Thermonuclear Experimental Reactor (ITER) and the China Fusion Engineering Test Reactor, achieving high-performance and steady-state plasmas for magnetically confined fusion (MCF) is one of the main goals. Therefore, it is necessary to sustain stable driven currents (noninductive), which mainly include the external auxiliary driven currents (such as those from neutral beam injection and radio-frequency wave-driven currents) and the bootstrap current (BC). The BC physically originates from the banana orbits of trapped electrons in the presence of a pressure gradient. It is similar to the classical diamagnetic drift current due to particle gyration, while it flows along the magnetic field lines. Through collisions with trapped particles and passing particles, the momentum is transferred to the passing electrons. The result of these collisions is a so-called BC carried by passing electrons, which is proportional to the gradients of density and temperature. If the power of an external auxiliary driven current is high, it will greatly increase the cost of a tokamak fusion reactor. Therefore, it is important to find ways to increase the fraction of BC in order to reduce the power requirements needed by the external auxiliary driven currents for ITER steady-state operation.

However, a high fraction of BC will trigger NTMs that are driven by the perturbed helical BC due to pressure flattening across the island, even if the TM is stable. TMs (including NTMs) can have a significant negative impact on the performance of MCF plasmas [2]. They can increase the local radial transport, degrade plasma confinement and lead to disruption in high-β plasmas ($\beta = 8 \pi p/B_{0}^{2}$, where *p* and *B*_0_ are the plasma pressure and magnetic field, respectively), resulting in a limit on the maximum achievable β [3]. Hence, understanding the physics and control of TMs is one of the critical issues of future MCF devices [3].

The physics of energetic particles (EPs) is particularly relevant to the dynamics of MCF plasmas where EPs are inevitably produced in the burning plasma or during auxiliary heating, such as alpha particles (the products of deuterium–tritium fusion). Not only do the transport and confinement of EPs affect machine performance, but also relatively small alpha losses can damage the machine’s first wall owing to the large energy carried by alpha particles and must be avoided. On the other hand, EPs can interact strongly with the background plasma and give rise to lots of new phenomena. They can not only affect existing instabilities, but also drive some new instabilities, which will have an impact on plasma performance. In turn, these instabilities can affect the loss and redistribution of EPs. For example, the driving of Alfvén eigenmodes or other modes (e.g. fishbone modes) by EPs will affect the transport of these particles and degrade plasma performance [4–6]. Burning plasmas constitute complex self-organized systems, posing a great challenge for both experimental and theoretical plasma physics. There are a vast class of problems involved, ranging from basic science to applied physics. Among these, the physics of EPs is one of the critical problems [7].

There are significant interactions between TMs and EPs, as shown by many experiments and theoretical studies on tokamak plasmas. These interactions are reflected in two ways: (1) EPs affect the stability and evolution of TMs; (2) TMs lead in turn to the loss and redistribution of EPs. Here, we simply review the history of this topic.

Many experiments in MCF devices have found significant effects of EPs on TMs [8–12]. In DIII-D [8] and ASDEX-U [9], it was shown that the onset threshold of NTMs is affected by neutral beam injection (NBI): the onset threshold increases with increasing co-NBI power, with different results for counter-NBI. In the National Spherical Torus Experiment (NSTX) using a spherical torus, the onset threshold of NTMs increases with increasing co-NBI power [10]. It has been suggested that toroidal rotation effects are involved, but they fail to explain those experimental results where the effects of EPs are significant. In the Madison Symmetric Torus (MST) with a reversed field pinch, it was found that the amplitude of TMs is greatly reduced (by up to 60%) by co-NBI [11]. An experiment in DIII-D has shown that the effects of energetic ions (EIs) on TMs are weak for a large island width [12].

Some theoretical and simulation studies have also been devoted to this topic [13–26]. In 1989, Hegna and Bhattacharjee [13] showed that EIs have stabilizing effects on nonlinear TMs by their interaction with the island region. They found that the current in an MI generated by magnetic drift of EIs depended on the density gradient of these ions outside the rational surface and increased the stability of nonlinear TMs. However, they did not take account of the effect of averaging over the orbital width of the EIs, and thus the influence of the EIs on stability was over-estimated. In 2009, a simulation by Takahashi *et al.* [14] showed that EIs can reduce the growth rate of TMs through interaction with the ideal outer region. In 2011, Cai *et al.* [15] analyzed the effects of circulating EIs (CEIs) on TMs and pointed out that EIs interact mainly with the ideal outer region of TMs, because the orbital width of the EIs is much larger than the island width. They also presented a instability criterion of TMs, taking account of the effects of CEIs. These effects depend on the direction of motion of the CEIs. In 2012, Cai and Fu [16] studied the effects of EIs on TMs using a global kinetic/magnetohydrodynamics (kinetic/MHD) hybrid simulation in which the dependencies of kinetic effects on EI beta, gyroradius and injection speed were systematically taken into account, and the results agreed in large part with previous analytical results for the kinetic effects of circulating particles. They also found that the effect of trapped EIs (TEIs) on TMs was much more destabilizing compared with that of counter-circulating particles at the same beta value. A new fishbone-like mode was also found. Subsequently, the effects of TEIs on the instability criterion of TMs were investigated by Halfmoon and Brennan [18] and Zhang *et al.* [19]. The effects of EPs on NTMs through an uncompensated cross-field current due to a large energetic-ion orbit were studied by Cai [20], who predicted that the effects are most significant in plasmas with weak magnetic shear. The effects of toroidal rotation due to NBI on TMs were studied by Cai *et al.* [21,22], who provided a qualitative explanation for the differences in experimental results between DIII-D and ASDEX-U as being due to the combined effects of EPs and rotation.

In addition to the above nonresonant wave–particle effects, TMs can resonate with EPs. A rapid frequency chirping of NTMs by EPs has been observed on the Tokamak Fusion Test Reactor (TFTR) [27], ASDEX-U [28], EAST [29], HL-2A [30] and DIII-D [31]. In TFTR, rapid frequency chirping up and down during the evolution of NTMs has frequently been observed [27]. Accompanying each chirping, there is a measurable reduction in the neutron rate, with the largest being of the order of 1%. It has been suggested that the chirps are associated with redistribution or loss of EPs. In the case of ASDEX-U, it has been suggested that by analogy with the occurrence of fishbone-like modes, the frequency chirping of the TM is caused by resonant interaction with EPs [28], with EPs transiently impressing their precession frequency on the pre-existing magnetic perturbation of the TM. In HL-2A, a frequency chirping of TMs within ∼1 ms is found [30], which only occurs while the TM rotation direction changes from electron to ion diamagnetic drift. In DIII-D, it was found that the mode frequency first jumps up from the steady NTM frequency, then chirps down, and finally returns to the steady NTM frequency within ∼1 ms [31]. During each chirp, a measurable reduction in the neutron rate is also found, with the largest drop at each chirp being of the order of 1%. In 2000, Marchenko and Lutsenko [32] proposed an explanation of the experimental results in ASDEX-U, according to which the resonant interaction between TEIs and TM provided an additional toroidal torque to accelerate the MI and thus drive limit-cycle modulations of the TM frequency and amplitude. However, the chirping time given in [32] is much longer than that observed experimentally. Recently, in contrast to the particle model used in [32], a drift kinetic theory is used to explain this phenomena [33] based on the DIII-D experiment [31]. The calculated chirping time and predicted island propagation are well consistent with DIII-D experimental results. Recent simulation and analytical studies have led to the proposal that the frequency chirping is due to an energetic-particle-driven mode. According to the simulation, this mode is driven by passing EIs [34]. The analytical results suggest that the mode is driven by TEIs [35], since the effect of resonance between passing EIs and the mode can be neglected in the absence of a finite-orbit effect.

TMs can in turn have an impact on EPs transport. Dramatic losses of EPs due to TMs have been found and explored both experimentally and theoretically [12,36–50]. In TFTR, the loss of alpha particles can be up to five times the loss in the absence of strong MHD instabilities [36]. In DIII-D, the current driven by NBI is less than the classical prediction (by up to 80%) in the presence of TMs [38]. In NSTX, it has been found that the loss of EIs due to TMs reduces the toroidal beta by 7% and the driven rotation by 20% [39]. In ASDEX-U, the NTM-induced loss of EIs can be of the same order as the prompt loss, up to 4 × 10^14^ ions s^−1^ cm^−2^ [40]. It has been shown that tearing-mode-induced transport of EIs causes dramatic changes in the spatial profiles of EIs in DIII-D [12]. In a reversed field pinch like MST, it has also been shown that the outward transport of EP is enhanced by core TMs [45]. Hence, the transport and confinement of EPs due to TMs can be significant.

The mechanism of transport of EPs due to TMs differs from that induced by resonance with high-frequency modes, since resonance between EPs and TMs is barely possible, owing to the very low frequency of TMs. In addition to prompt loss of EPs, the transport mechanisms of both circulating and trapped EPs have been classified. For circulating EPs (CEPs), in the presence of an MI, so-called drift islands are formed in phase space [37]. Owing to the poloidal dependence of magnetic drift, in addition to the main drift island, a series of sideband drift islands are also formed. The width of the main drift island is nearly identical to the width of the MI, while the sideband drift islands are smaller but have widths that are proportional to the MI width and are related to the particle energy. When the MI width and particle energy are sufficiently large, the drift islands will overlap, leading to stochastic orbits that will greatly increase the loss of EPs. Even without stochastic orbit formation, however, the drift islands will also have significant effects on the profiles of EPs [41].

For trapped energetic particles (TEPs), perturbation of TMs plays a similar role to the ripple-field-induced loss of TEPs by stochastic diffusion and ripple well trapping. Magnetic perturbation gives rise to excursions near the turning points of the banana orbits of EPs. The radial shift of the turning points causes expulsion of TEPs. In addition to this mechanism, resonance-induced loss is caused by a particular resonance between EPs and TMs. As the particle energy increases, the precession frequency of trapped particles will beat with the bounce frequency owing to their different dependence on energy [44]. Then, a resonance will occur even in the limit of vanishing mode frequency; owing to this resonance, the orbital average of the radial drift due to the magnetic perturbation is finite. The pile up of the radial shift in every bounce period will lead to the expulsion of TEPs. On the other hand, fast frequency chirping during TMs has been found in some experiments, as mentioned above. This frequency chirping indicates that a strong resonance occurs between EPs and TMs. This resonance will also cause loss of EPs [27–31].

In this review, we consider only the standard orbits of EPs in axisymmetric toroidal configurations, under the assumption that the radial excursion of an orbit guiding center across a given magnetic flux surface is small compared with the mean distance of this surface from the magnetic axis. With this assumption, standard orbits are classified into two groups: passing and trapped orbits. If this assumption or that of an axisymmetric toroidal configuration is not satisfied, the orbits of EPs will be distorted into nonstandard orbits [42].

In the following sections we respectively review the basic physics of TMs; the sources of EPs and the gyrokinetic equations describing the dynamics of EPs; the influence of EPs on TMs in five parts; and the influence of TMs on EP transport. In the final section, conclusions, a discussion and an outlook are given.

## BASIC PHYSICS OF TMs

In this section, we simply review the basic physics of TMs. The parallel Ohm’s law and momentum equation are

(1) \begin{eqnarray*} E_{\parallel } = \eta J_{\parallel }, \end{eqnarray*}

(2) \begin{eqnarray*} \mathbf {B} \cdot \nabla \frac{J_{\parallel }}{B} + \nabla \cdot \frac{\mathbf {J}_{\perp }}{B} = 0. \end{eqnarray*}

Here, $E_{\parallel } = - \mathbf {b} \cdot \nabla \phi - (1/c) \partial A_{\parallel } / \partial t$ is the parallel electric field, where φ is the electrostatic potential and *A*_∥_ is the parallel magnetic vector potential; $\mathbf {B}$ is the magnetic field (with $\mathbf {b} = \mathbf {B}/B$); η is the resistivity; *J*_∥_ is the parallel current density; and $\mathbf {J}_{\perp }$ is the perpendicular current density, resulting from the (neoclassical) polarization current and diamagnetic drift current.

In the absence of resistivity, an ideal MHD eigenequation can be obtained from Equations (1) and (2). It is known that this equation is singular at the rational surface, i.e. some physics must be included to remove this singularity, such as the finite-Larmor-radius effect or resistivity. Including the effects of resistivity leads to resistive instabilities. To solve Equations (1) and (2), a boundary layer approach is adopted, namely, the radial space is separated into a resistive layer and outer region where the resistive and ideal MHD equations are respectively solved. All the radial, poloidal and toroidal angular derivatives of the perturbed quantities are assumed to be of order unity, *L*_eq_ ∂ln δ*f*/∂*r* ∼ ∂ln δ*f*/∂θ ∼ ∂ln δ*f*/∂ζ ∼ 1, except the radial derivative in the resistive layer is assumed to be large, *L*_eq_ ∂ln δ*f*/∂*r* ≫ 1. Here, δ*f* denotes the perturbed quantities, *L*_eq_ is the typical scale size at equilibrium, *r* is the radius, and θ and ζ are the poloidal and toroidal angles, respectively. Then, by matching the solutions in the outer region and resistive layer (island region for the nonlinear phase), the generalized dispersion relation of the TM can be obtained as

(3) \begin{eqnarray*} \Delta ^{\prime } = \frac{8 \pi R_{0}}{c} \frac{1}{\delta \hat{\psi }} \int _{0^{-}}^{0^{+}}{\rm d} x \oint \frac{{\rm d} \xi }{2 \pi }\, \delta J_{\parallel } \exp {(i \xi )},\\ \end{eqnarray*}

where *x* = *r* − *r_s_*, with *r_s_* the location of the rational surface, and *R*_0_ is the major radius at the magnetic axis. Here, $\Delta ^{\prime } = (\partial {\ln {\delta \psi }}/\partial r)|_{r_{s}^{-}}^{r_{s}^{+}}$ is generally a complex value and is determined by the solution in the outer region. The term on the right-hand side of Equation (3) is determined by the solution in the resistive layer, where δ*J*_∥_ ∼ ∂^2^δψ/∂*x*^2^ is the perturbed parallel current density. As the key measure of the plasma free energy, Δ′ is essential to the stability of classical TMs and to the onset and evolution of TMs. The real part $\Delta _{c}^{\prime }$ of Δ′ provides the instability criterion for TMs: if $\Delta ^{\prime }_{c} >0$, the TM is unstable. The related equation including the imaginary part $\Delta _{s}^{\prime }$ describes the evolution of the frequency of the TM.

The magnetic field for the TM is given by

(4) \begin{eqnarray*} \mathbf {B} = I \nabla \zeta + \nabla \zeta \times \nabla (\psi + \delta \psi ), \end{eqnarray*}

where an axisymmetric toroidal geometry is assumed; *I*/*R* is the toroidal magnetic field, and ψ and δψ are the equilibrium and perturbed poloidal magnetic flux, respectively. Here, δ*B*_∥_ is neglected, i.e. only the ‘slow’ MHD time scale given by the shear Alfvén wave time scale *R*/*v_A_* is considered [51]. We have $\delta \psi = \delta \hat{\psi } (0,t) \cos {\xi }$ with ξ = *m*θ − *n*ζ − ∫^*t*^ω(*t*′) d*t*′, where *m* and *n* are the poloidal and toroidal mode numbers, respectively. We denote by ω the rotation frequency of TMs relative to the plasma. Here, a single helicity is considered, and the familiar constant-$\delta \hat{\psi }$ approximation in the resistive layer for TMs is adopted. Then, Equation (3) can be rewritten as

(5) \begin{eqnarray*} \Delta ^{\prime }_{c} &=& \frac{8 \pi R_{0}}{c} \frac{1}{\delta \hat{\psi }} \int _{0^{-}}^{0^{+}}{\rm d} x\oint \frac{{\rm d} \xi }{2 \pi }\,\\ &&\times \, ( \delta J_{\parallel ,R} \cos {\xi }- \delta J_{\parallel ,I} \sin {\xi } ), \end{eqnarray*}

(6) \begin{eqnarray*} \Delta ^{\prime }_{s} &=& \frac{8 \pi R_{0}}{c} \frac{1}{\delta \hat{\psi }} \int _{0^{-}}^{0^{+}}{\rm d} x \oint \frac{{\rm d} \xi }{2 \pi }\,\\ &&\times \, ( \delta J_{\parallel ,R} \sin {\xi }+ \delta J_{\parallel ,I} \cos {\xi } ), \end{eqnarray*}

where δ*J*_∥, *R*_ and δ*J*_∥, *I*_ are the real and imaginary parts of δ*J*_∥_ (which is calculated in the island region), respectively. Here *J*_∥, *I*_ results from wave–particle resonance or wave–wave coupling. Expression (5) is used to calculate the growth rate of TMs in the linear phase, and to determine the evolution of the MI width in the nonlinear phase. Expression (6) is used to calculate the rotation frequency in both the linear and nonlinear phases.

As indicated above, Δ′ is determined by the ideal MHD equation with a boundary condition in the outer region where the ideal MHD approximation is made, and the nonlinear effect and the inertial term can be neglected. Then, the classical ideal equation for the TM in the outer region can be obtained from Equation (2) as

(7) \begin{eqnarray*} \mathbf {B}_{0} \cdot \nabla \frac{\delta J_{\parallel }}{B_{0}} + \delta \mathbf {B} \cdot \nabla \frac{J_{\parallel ,0}}{B_{0}} = 0, \end{eqnarray*}

where the contribution of pressure is ignored. Then, Equation (7) can be obtained as

(8) \begin{eqnarray*} \bigg ( \frac{m}{n} &-& q \bigg ) \frac{\varepsilon }{q} \bigg [ \frac{d}{d r} \bigg ( r \frac{d \delta \hat{\psi }_{\parallel }}{d r} \bigg ) - \frac{m^{2}}{r} \delta \hat{\psi }_{\parallel } \bigg ]\\ && - \frac{m}{n} \frac{d J_{\parallel 0}}{d r} \delta \hat{\psi }_{\parallel } = 0, \end{eqnarray*}

where the variables are normalized as $\mathbf {r} \rightarrow a \mathbf {r}$ and $\mathbf {B} \rightarrow B_{0} \mathbf {B}$, and ε = *r*/*R*_0_. Then, the instability criterion with plasma boundary condition can be solved as [15]

(9) \begin{eqnarray*} \Delta ^{\prime }_{c} = - \frac{\pi \alpha _{0}}{r_{s}} \cot \big [ \pi \big ( \sqrt{m^{2} + \alpha _{0}} - m \big ) \big ], \end{eqnarray*}

where

(10) \begin{eqnarray*} \alpha _{0} = - q^{2}(r_{s}) \bigg [ \varepsilon \frac{{\rm d} q(r_{s})}{{\rm d} r} \bigg ]^{-1} \frac{{\rm d} J_{\parallel 0} }{{\rm d} r}. \end{eqnarray*}

Equation (9) is valid for 0 < α_0_ < 2*m* + 1, since it is derived from the leading-order expansion near the resistive layer for the solution of Equation (8). To obtain the full solution, it is necessary to resort to a numerical approach, such as a shooting method. Now, the current in the resistive layer on the right-hand side of Equation (3) must be solved.

For the linear TM, the linearized equations in the resistive layer can be obtained from Equations (1) and (2) as

(11) \begin{eqnarray*} \hat{\gamma } \delta \hat{\psi }(0) + \frac{n s}{r_{s}} x \delta \hat{\phi } = \hat{\eta } \frac{{\rm d}^{2} \delta \hat{\phi }}{{\rm d} x^{2}}, \end{eqnarray*}

(12) \begin{eqnarray*} \hat{\rho }_{s} \hat{\gamma } \frac{{\rm d}^{2} \delta \hat{\phi }}{{\rm d} x^{2}} = \frac{n s}{r_{s}} x \frac{{\rm d}^{2} \delta \hat{\psi }}{{\rm d} x^{2}}. \end{eqnarray*}

It is assumed here that the fluid velocity $\mathbf {v} \sim \mathbf {v}_{E}$, where $\mathbf {v}_{E}$ is the $\mathbf {E} \times \mathbf {B}$ drift, and the diamagnetic drift current is not considered. The variables are normalized as *r* → *ar, t* → τ_*A*_*t*, ρ → ρ(0)ρ, δϕ → *B*(0)*a*^2^δϕ, δψ → *B*(0)*a*^2^δψ, $\hat{\eta } = \tau _{R}/\tau _{A}$, where τ_*R*_ = μ_0_*a*^2^/η is the resistivity diffusion time, τ_*A*_ = *R*/*v_A_* is the Alfvén time, $v_{A} = B_{0}/ \sqrt{4 \pi \rho _{0}}$ and ω_*A*_ = 1/τ_*A*_ is the Alfvén frequency. *s* is the magnetic shear. Coupling between pressure and magnetic curvature is ignored. Here, the parameters are expanded near the rational surface *r_s_* as *x* = *r* − *r_s_* ∼ *O*(δ_res_), where δ_res_ is the resistive layer width of the linear TM. Equations (11) and (12) can then be solved. Substituting the solutions into the general dispersion relation (3), one can obtain (52)

(13) \begin{eqnarray*} \Delta ^{\prime }_{c} & =& \frac{1}{\delta \psi (0)} \int _{0^{-}}^{0^{+}} \frac{\partial ^{2} \delta \psi }{\partial x^{2}}\, {\rm d} x \\ &=& \frac{2 \pi \Gamma (3/4)}{\Gamma {(1/4)}} \bigg (\frac{n s}{r_{s}}\bigg )^{-1/2} \hat{\rho }_{s}^{1/4} \hat{\gamma }^{5/4} \hat{\eta }^{-3/4},\\ \end{eqnarray*}

where the rotation frequency of the TM is ignored. Then, the growth rate of the classical TM can be obtained as $\hat{\gamma } = \hat{\Delta }^{\prime 4/5} \hat{\eta }^{3/5}$ [52–54], where $\hat{\Delta }^{\prime } = (n s /r_{s})^{1/2} [2 \pi \Gamma (3/4)/\Gamma (1/4)]^{-1} \hat{\rho _{s}}^{-1/4} \Delta ^{\prime }$.

For nonlinear TMs, the fundamental ordering δ_res_ ≪ *w* ≪ *a* is satisfied. With the contributions from the polarization current and diamagnetic drift current ignored, Equation (2) gives $\mathbf {B} \cdot \nabla J_{\parallel }/B =0$. Thus, one can obtain *J*_∥_ = *J*_∥_(Ω) with Ω = 2*x*^2^/*w*^2^ − cos ξ the magnetic flux surface with an island, where $w=2 \sqrt{q_{s} \delta \hat{\psi }/(q_{s}^{\prime } \psi _{s}^{\prime })}$ is the island width (the prime denotes the derivative with respect to *r*, and the subscript *s* indicates values at *r_s_*). Then, combined with Equation (1), *J*_∥_ = −〈∂δψ/∂*t*〉/η, where $\langle \cdot \rangle _{\Omega } = \oint {\rm d} \xi /(2 \pi )\, (\cdot )/\sqrt{\Omega + \cos {\xi }}$. Substituting this into Equation (5) gives the Rutherford evolution equation [52,55] as

(14) \begin{eqnarray*} \frac{8 \pi }{\eta c^{2}} I_{1} \frac{{\rm d} w}{{\rm d} t} = \Delta ^{\prime }_{c}, \end{eqnarray*}

where *I*_1_ ≃ 0.83. If the BC and the neoclassical polarization current are included, Equation (14) can be extended to give the generalized Rutherford evolution equation for an MI of NTMs as [20]

(15) \begin{eqnarray*} \frac{8 \pi }{\eta c^{2}} I_{1} \frac{{\rm d} w}{{\rm d} t} = \Delta ^{\prime }_{c} + \Delta _{b}^{\prime } + \Delta _{\pi }^{\prime } , \end{eqnarray*}

where $\Delta _{b}^{\prime }$ and $\Delta _{\pi }^{\prime }$ [20] result from the contributions of the BC and the neoclassical polarization current, respectively, as

(16) \begin{eqnarray*} \Delta ^{\prime }_{b}= G_{1} \sqrt{\epsilon _{s}} \, \frac{r_{s}}{s L_{n}} \frac{\beta _{\theta i}}{w}, \end{eqnarray*}

(17) \begin{eqnarray*} \Delta ^{\prime }_{\pi } = - 1.64 \epsilon _{s}^{3/2} G_{2} \frac{r_{s}^2}{s^2 L_{n_i}^{2}} \frac{\rho _{\theta i}^2}{w^{2}} \frac{\beta _{\theta i}}{w} \frac{\omega (\omega - \omega _{*i})}{\omega _{*i}^2}.\\ \end{eqnarray*}

The numerical coefficients *I*_1_ ≃ 0.83, *G*_1_ ≃ 2.31 [56], *G*_2_ ≃ 1.42 and *G*_3_ ≃ 1.58, ω_*i_ = *cm*/(*n*_*i*0_*eq*)d*p*_*i*0_/dψ is the ion diamagnetic current, *n*_*i*0_ is the density of thermal ions, ρ_θ*i*_ is the ion poloidal Larmor radius, $\beta _{\theta i} = 8 \pi p_{i}/B^{2}_{0}$ and $L_{n_{i}}$ is the scale length of ion density. The frequency ω is determined by the torque balance, which remains a matter of debate. The subscript *s* indicates values at the rational surface *r_s_*. Here, the effect of finite transport on the BC contribution is not considered in $\Delta ^{\prime }_{b}$ [56–58]. From expressions (5) and (15), it can be seen that there are two ways to affect the dynamics of the island width. One is to change the value of $\Delta ^{\prime }_{c}$ by changing the equilibrium parallel current, the other is to change the parallel current in the island. Actually, electron cyclotron current drive (ECCD) is used to control NTMs [59–61]. The dominant driving mechanism of NTMs is the loss of BC due to the flattened pressure profile within the island. ECCD then provides an additional current to compensate for the missing BC in the island. This will also affect $\Delta ^{\prime }_{c}$. Similarly, the influence of EPs on NTMs is reflected in the effects on both Δ′ and *J*_∥_ in the island.

## GYROKINETIC EQUATIONS OF EPs

EPs are abundant in MCF plasmas, including EIs and energetic electrons. There are three main sources of EIs: (i) fusion reactions such as _1_D^2^ + _1_T^3^ → _2_He^4^ (3.5 MeV) + *n* (14.1 MeV) (which is preferred to other reactions), (ii) NBI and (iii) resonance heating with radio-frequency waves, such as ion cyclotron resonance heating (ICRH) and lower hybrid current drive (LHCD). For details, see [7].

- In a burning plasma, a large population of alpha particles is generated by DT fusion reaction, as shown in experiments in TFTR and JET. The parameters of the EIs in different tokamaks can be found in [7], where it can be seen that the EI density *n_h_* and β_*h*_ ($\beta = 8 \pi p_{h}/B_{0}^{2}$, *p_h_* is the pressure of EIs) are of the order of 1%. It should be noted that the angular distribution of alpha particles from fusion reactions is nearly isotropic, while the spatial distribution is centrally peaked.
- With NBI, an injected energetic atom can be ionized by electron impact ionization reactions. The ionized particles are first slowed down mainly by collisions with background electrons and then scattered by collisions with ions. The angular distribution of EIs resulting from NBI is anisotropic and depends on the injection angle, while their spatial distribution depends on injection energy and plasma density.
- ICRH heats a plasma initially by resonance heating of ions. During this process, ions are accelerated. The angular distribution of EIs produced by ICRH is anisotropic and perpendicular to the background magnetic field, while their spatial distribution is peaked near the resonance layer. LHCD is a candidate for current profile control in ITER, while it is the main current-driven source in EAST. The angular distribution of EIs from LHCD is bi-Maxwellian, while their spatial distribution depends on the plasma density profile.

The large population of EIs produced in these various ways and the large amount of energy that they carry result in a number of new plasma phenomena. In addition, there are energetic electrons that are mainly from electrons accelerated by radio-frequency waves in the electron cyclotron range of frequency and runaway electrons produced during plasma disruption in tokamaks. These also have a significant impact on plasma instabilities.

When considering the physics of EPs in an MCF plasma, it is generally assumed that the plasma consists of two components: (i) a core plasma with thermal ions and electrons and (ii) EPs. The EPs can be described using gyrokinetic or drift kinetic theory owing to the large energy that they carry. For the core plasma, thermal ions or electrons are described by kinetic theory or by MHD, depending on the particular physical processes of interest. For example, the kinetic effect of thermal ions could be important in the low collision regime [62] or if the orbit width of thermal ions is comparable to the MI width [58]. In this review, the core plasma is described by MHD for simplicity, since we focus on the interaction between EPs and NTMs. The basic gyrokinetic equations for describe the behavior of EPs are [4]

(18) \begin{eqnarray*} \delta f &=& e^{-\boldsymbol {\rho } \cdot \nabla } \delta g + \frac{e}{m_{h}} \frac{\partial F_{0}}{\partial E} \delta \phi +\, \frac{e}{m_{h}} \frac{1}{B_{0}} \frac{\partial F_{0}}{\partial \mu }\\ &&\times \, ( \delta L - e^{-\boldsymbol {\rho } \cdot \nabla } \langle \delta L_{g}\rangle _{g} ), \end{eqnarray*}

(19) \begin{eqnarray*} \frac{{\rm d} \delta g}{{\rm d} t} &=& - \bigg (\frac{e}{m_{h}} \frac{\partial F_{0}}{\partial E} \frac{\partial }{\partial t}- \frac{c}{B_{0}} \mathbf {b} \!\times \! \nabla F_{0} \cdot\! \nabla \bigg ) \langle \delta L_{g}\rangle _{g}\\ && - \frac{c}{B_{0}} \mathbf {b} \times \nabla \langle \delta L_{g}\rangle _{g} \cdot \nabla \delta g. \end{eqnarray*}

Here, $\boldsymbol {\rho } = \omega _{c}^{-1} \mathbf {b}_{0} \times \mathbf {v}$ is the gyroradius, where ω_*c*_ is the cyclotron frequency and $\mathbf {b}_{0} = \mathbf {B}_{0}/B_{0}$ is the direction of the equilibrium magnetic field; *E* = *v*^2^/2 is the energy per unit mass; μ is the magnetic moment; δ*L* = δφ − (*v*_∥_/*c*)δ*A*_∥_ and $\delta L_{g} = e^{\boldsymbol {\rho } \cdot \nabla } \delta L$, where δφ is the perturbed electrostatic potential and $\delta \mathbf {A} = \delta A_{\parallel } \mathbf {b}_{0}$ is the perturbed magnetic vector potential, with δ*A*_∥_ = −δψ/*R*; 〈·〉_*g*_ denotes the gyrophase average;

$$\begin{equation*}
\frac{{\rm d}}{{\rm d} t }= \frac{\partial }{\partial t} + v_{\parallel } \mathbf {b}_{0} \cdot \nabla + \mathbf {v}_{d} \cdot \nabla
\end{equation*}$$

is a linear operator, with $\mathbf {v}_{d} = \mathbf {b}_{0} \times (\mu \nabla B_{0} + v_{\parallel }^{2} \boldsymbol {\kappa })/\omega _{c}$, where $\boldsymbol {\kappa } = \mathbf {b}_{0} \cdot \nabla {\mathbf {b}}_{0}$ is the magnetic curvature; δ*f* is the perturbed distribution and *F*_0_ = *F*_0_(*p*_φ_, *E*, μ) is the equilibrium distribution, where *p*_φ_ = *e*ψ + *v*_∥_*RB*_ζ_/*B* is the toroidal canonical momentum. In Equations (18) and (19), δ*B*_∥_ is neglected for NTMs, i.e. only the ‘slow’ MHD time scale given by the shear Alfvén wave time scale *R*/*v_A_* is considered [51]. The final term on the right-hand side of Equation (19) is a nonlinear term. Equations (18) and (19) are the basic gyrokinetic equations for describing the behavior of EPs.

## INFLUENCE OF EPs ON TMs

From the dispersion relation (5), it can be seen that EPs can influence TMs through interaction with the outer region and resistive layer (island region in the nonlinear phase). In this review, we assume that the orbital width of the EPs is much larger than the island width. This is always the case for the linear phase, the early nonlinear phase and the onset threshold of NTMs. For typical tokamak parameters, the seed island of NTMs is about 1 cm in size [3]. It is typical of the thermal ion banana width. The ion orbit width of EIs is much larger than the thermal ion banana width and the seed island width. In this case, the orbits of EPs are mainly in the outer region, i.e. EPs mainly interact directly with TMs in the outer region. In the island region, owing to quasineutrality, EPs will provide an uncompensated current because their response is significantly reduced by the orbital averaging effect in the limit of large orbital width. Next, we review the physics in the outer region and island region, respectively.

### Influence of CEPs in the outer region

In this subsection, we focus on the interaction between CEPs and TMs in the outer region, based on [15,16]. To understand the underlying physics, a heuristic interpretation will be given [15]. In the case of classical TMs without EPs, $\mathbf {B} \cdot \nabla J_{\parallel }/B =0$ for a low-β plasma in the outer region, where the diamagnetic current due to the pressure of background plasma is ignored. Then, one obtains the flux function *J*_∥_ = *J*_∥_(Ψ), where Ψ = *Q*(ψ) + δψ, with d*Q*/dψ = 1 − *q*/*q_s_* (*q_s_* = *m*/*n* is the value of *q* at the rational surface), satisfies $\mathbf {B} \cdot \nabla \Psi =0$. Thus, the perturbed parallel current density δ*J*_∥_ = (d*J*_∥, 0_/dψ)(1 − *q*/*q_s_*)^−1^δψ. This is the ideal MHD equation for the TM in the outer region, corresponding to Equation (7). When EPs are included, the total current density *J*_∥_ = *J*_∥, *c*_ + *J*_∥, *h*_, where *J*_∥, *c*_ and *J*_∥, *h*_ are the current densities of the background plasma and EPs, respectively. The background plasma current density *J*_∥, *c*_ = *J*_∥, *c*_(Ψ) is still a flux function, but *J*_∥, *h*_ is not, owing to its large drift orbit, which satisfies $\mathbf {B} \cdot \nabla J_{\parallel }/B + \nabla \cdot \mathbf {J}_{\perp h}=0$, where $\mathbf {J}_{\perp h}$ is the diamagnetic drift current density of EPs. For EPs, at locations in the particle drift orbit, *J*_∥, *h*_ = *J*_∥, *h*_(Ψ_*d*_) is a drift flux function, where Ψ_*d*_ = *Q_d_*(ψ_*d*_) + 〈δψ〉_*b*_(ψ_*d*_), d*Q_d_*/dψ_*d*_ = 1 − *q*(ψ_*d*_)/*q_s_* and ψ_*d*_ = ψ − *v*_∥_*I*/ω_*c*_ + 〈*v*_∥_*I*/ω_*c*_〉_*b*_, where 〈·〉_*b*_ = ∮(·) d*t*/τ_*b*_ denotes the orbital average (τ_*b*_ is the period of the particle orbit). Then, transforming to spatial coordinates and taking the orbital average, one obtains 〈δ*J*_∥, *h*_〉_*b*_ = (d*J*_∥, *h*0_/d*Q_d_*)*a*^2^δψ(ψ), where *a*^2^ ≤ 1 represents the effect of orbital averaging. If the orbital width is much larger than the island width then *a*^2^ ∼ 0, while if it is much smaller than the island width then *a*^2^ ∼ 1. Thus, the total perturbed parallel current density can be written as δ*J*_∥_ = [(d*J*_∥, 0_/dψ)δψ − (d*J*_∥, *h*0_/dψ)(1 − *a*^2^)δψ](1 − *q*/*q_s_*)^−1^. The direction of the current density driven by co-CEI is the same as that of the total current density, i.e. the signs of *J*_∥, *h*0_ and *J*_∥, 0_ are the same. Then d*J*_∥, *h*0_/dψ and d*J*_∥, 0_/dψ have same sign for monotonic profiles. Thus, it can be found that co-CEIs tend to reduce the total perturbed current density, i.e. it plays a stabilizing role. On the other hand, counter-CEIs play a destabilizing role. This physical picture can also be applied to the case of energetic electrons. In this physical picture, it can be imagined that EPs, like a steel wire, have a stiff response to perturbations, owing to their high energy compared with thermal particles. Thus, they are hardly affected by plasma perturbations. This is reflected in the physical picture and associated expressions presented above. Next, a more detailed description will be given, based on this physical interpretation and on the presentation in [15].

In the outer region, based on the quasineutrality condition, the linearized equation for the perturbed parallel current density is

(20) \begin{eqnarray*} \mathbf {B}_{0} &\cdot & \nabla \frac{\delta J_{\parallel }}{B_{0}} + \delta \mathbf {B} \cdot \nabla \frac{J_{\parallel ,0}}{B_{0}} + \frac{2 c \mathbf {b}_{0} \times \boldsymbol {\kappa }}{B_{0}} \cdot \nabla \delta p_{c}\\ & +& \frac{c \mathbf {b}_{0} \times \boldsymbol {\kappa }}{B_{0}} \cdot \nabla ( \delta p_{\parallel ,h} + \delta p_{\perp ,h} ) = 0, \end{eqnarray*}

where δ*p_c_* is the perturbed core plasma pressure, $(\delta p_{\parallel ,h}, \delta p_{\perp ,h}) = \int d^{3} v \, (v_{\parallel }^{2}, \mu B) \delta f_{h}$, δ*f_h_* is the perturbed distribution of EIs. Next, the perturbed distribution of EIs must be derived. For EPs, the linearized drift kinetic equations can be obtained from Equations (18) and (19) as

(21) \begin{eqnarray*} \delta f_{h} = \frac{e \delta \phi }{m_{h}} \frac{\partial F_{h0}}{\partial E} + \delta g_{h}, \end{eqnarray*}

(22) \begin{eqnarray*} \frac{{\rm d} \delta g_{h}}{{\rm d} t} = \frac{i e}{ m_{h}} Q_{h} \bigg ( \delta \phi - \frac{v_{\parallel } \delta A_{\parallel } }{ c} \bigg ), \end{eqnarray*}

where *Q_h_* = (ω∂/∂*E* + ω_*h_)*F*_*h*0_ with $\omega _{*h} = - i (\mathbf {b} \times \nabla \ln F_{h0} / \omega _{ch}) \cdot \nabla$. Here, the finite-Larmor-radius effect is neglected, since the Larmor radius ρ_*c*_ = *v*_⊥_/ω_*c*_ is smaller than the orbital width (the drift orbit width for CEI is *qv*/ω_*c*_, and the banana width for TEI is $(q/\sqrt{\epsilon }) v/\omega _{c}$). Then, the effect of orbital averaging is larger than that of gyro-averaging. By introducing the transforms $\delta A_{\parallel } = -i c \mathbf {b} \cdot \nabla \delta \phi ^{r} / \omega$ and δ*g_h_* = −(*e*/*m_h_*ω)*Q_h_*δφ^*r*^ + δ*G*, δφ^*r*^ = δφ can be obtained based on the ideal MHD approximation δ*E*_∥_ = 0. Then, Equation (22) can be written as

(23) \begin{eqnarray*} \frac{{\rm d} \delta G_{h}}{{\rm d} t} = \delta H_{m}(r) e^{i m \theta - i n \zeta - i \omega t}, \end{eqnarray*}

(24) \begin{eqnarray*} \delta H_{m} = - \frac{e}{m_{h} \omega } Q v_{d} \bigg ( \frac{i m \cos {\theta }}{r} + i k_{r} \sin {\theta } \bigg ) \delta \hat{\phi },\\ \end{eqnarray*}

where the single helicity is considered for simplicity, and $v_{d} = (v_{\parallel }^{2} + v_{\perp }^{2}/2)/R \omega _{c}$ with *k_r_* = −*i*∂/∂*r* acting on the perturbation. To solve Equation (23), one can expand it by ordering and proceed as in [15]. One can also use the method of characteristics by integrating following the particle orbit as in [17,19]. For a CEI, the orbit is given by *r_d_* = *r* − ρ_∥, θ_, ρ_∥, θ_ = *w_b_*cos θ, θ = ω_*t*_*t*, α = *q*(*r_d_*)θ − ζ and ω_*t*_ = *v*_∥_/(*qR*). Transforming from coordinates (*r*, θ, ζ) to particle coordinates (*r_d_*, θ, α), expression (24) for δ*H_m_* becomes

(25) \begin{eqnarray*} \delta H_{m} &=& - i \frac{e}{2 m_{h} \omega } Q v_{d} \delta \hat{\phi }(r_{d}) e^{i n \alpha - i \omega t} \\ &&\times \, \sum _{l} \bigg [ \bigg ( \frac{m}{r} - i k_{r} \bigg ) e^{i (m - n q + 1 + l) \omega _{t} t } \\ &&+\, \bigg ( \frac{m}{r} - i k_{r} \bigg ) e^{i (m - n q - 1 + l) \omega _{t} t } \bigg ]i^{l} J_{l}(\lambda _{r}),\\ \end{eqnarray*}

where λ_*r*_ = *k_r_w_b_*. The effect of the coordinate transformation on the equilibrium parameters is ignored, since ∂ln *f*_0_/∂*r* ≪ *k_r_*. Near the rational surface, *k_r_w_b_* ∼ *O*(1), where *w_b_* is the orbital width of the EIs, the effect of a finite orbital width becomes important, since the instability criterion Δ′ is very sensitive to the behavior of eigenfunctions near the rational surface. By integrating Equation (23) using Equation (25), one can then obtain δ*G*(*r_d_*) in the particle coordinates. Transforming δ*G*(*r_d_*) back to the coordinates (*r*, θ, ζ) gives

(26) \begin{eqnarray*} \delta G (r, \theta ) &=& - \frac{e}{m_{h} \omega } Q v_{d} \delta \phi (r) \\ &&\times \,\sum _{l,l^{\prime }} \frac{i^{l+l^{\prime }-1} e^{i (l + l^{\prime }) \theta }}{(m-n q +l) \omega _{t} - \omega }\\ &&\times \, \bigg (\frac{m}{r} \frac{{\rm d} J_{l}}{{\rm d} \lambda _{r}} -i k_{r} \frac{l}{\lambda _{r}} J_{l} \bigg ) J_{-l^{\prime }}.\\ \end{eqnarray*}

Thus, the perturbed distribution of CEIs has been obtained. Then, by applying the integration ∮dθexp (−*im*θ + *in*ζ + *i*ω*t*)/2π to Equation (20) and using Equations (21), (22) and (26), one obtains

(27) \begin{eqnarray*} n \bigg ( \frac{m}{n} &-& q \bigg ) \frac{c}{4 \pi B_{0}} \bigg [ \frac{1}{r} \frac{d}{d r} \bigg ( r \frac{d \delta \hat{A}_{\parallel }}{d r} \bigg ) - \frac{m^{2}}{r^{2}} \delta \hat{A}_{\parallel } \bigg ] \\ &-&\, m \bigg ( \frac{\partial \psi }{\partial r} \bigg )^{-1} \frac{d}{d r} \bigg ( \frac{J_{\parallel 0}}{B_{0}} \bigg ) \delta \hat{A}_{\parallel } + i \delta K = 0 ,\\ \end{eqnarray*}

(28) \begin{eqnarray*} \delta K&=& \frac{e}{m_{h}} \frac{R_{0}}{B_{0}} r \bigg ( \frac{\partial \psi }{\partial r} \bigg )^{-1} \int d^{3} v \, \frac{v_{\parallel }^{2}}{\rho _{h}} Q_{h}\\ &&\times \, \left[1 - J_{0}^{2}(\lambda _{r})\right] \delta \hat{A}_{\parallel }(r), \end{eqnarray*}

where the terms involving the core pressure and the adiabatic part of the EI pressure, which are *O*(ϵ) compared with other terms, are neglected (ϵ = *r*/*R*_0_ is the inverse of the aspect ratio). Here, the adiabatic part of the EI pressure results from the perturbed distribution (ω_*h_*F*_*h*0_/ω)(*e*δφ/*m_h_*). It is assumed that |ω| ≪ |ω_*t*_| for TMs, i.e. the resonance effect is not considered here. For CEIs, |δ*p*_⊥_| ≪ |δ*p*_∥_|. In fact, Equation (27) is an integro-differential equation, since the expression of δ*K* is the integro equation. Note that Equation (28) is valid only near the rational surface, where $(\partial \delta \hat{A}_{\parallel } / \partial r)/ \delta \hat{A}_{\parallel } \gg 1$. In the region far from the rational surface, CEIs have only an adiabatic effect, which is similar to that in the core plasma. On the other hand, the instability criterion Δ′ is determined mainly by the behavior of $\delta \hat{A}_{\parallel }$ near the rational surface. Thus, we focus on the effects of CEIs near the rational surface in the outer region. Owing to the large orbital width, CEIs couple the regions λ_*r*_ ≫ 1 and λ_*d*_ ∼ *O*(1), and thus they play a nonlocal effect. This is similar to the effect of a finite thermal ion Larmor radius. For a complete calculation, Equations (27)–(28) can be solved numerically by iteration.

To proceed further, the slowing-down distribution for CEIs is chosen as $F_{h,0} = \sum _{\sigma } F_{h,0}^{\sigma }$, with $F_{h,0}^{\sigma } = (2^{3/2} \pi m_{h} B_{0} E_{m}^{\sigma })^{-1} p_{h}^{\sigma } E^{-3/2} \delta (\lambda ) H(E-E_{m})$, where σ denotes the direction of circulation of the CEIs (σ = + for co-CEIs and σ = − for counter-CEIs). One then obtains

(29) \begin{eqnarray*} \bigg ( \frac{m}{n} &-& q \bigg ) \frac{\varepsilon }{q} \bigg [ \frac{d}{d r} \bigg ( r \frac{d \delta \hat{A}_{\parallel }}{d r} \bigg ) - \frac{m^{2}}{r} \delta \hat{A}_{\parallel } \bigg ]\\ & -& \frac{m}{n} \bigg ( \frac{d J_{\parallel 0}}{d r} - \chi _{0} \sum _{\sigma } \frac{\sigma }{\rho _{hm}^{\sigma }} \frac{d \beta _{h}^{\sigma }}{d r} \bigg ) \delta \hat{A}_{\parallel } = 0,\\ \end{eqnarray*}

where $\rho _{hm}^{\sigma } = (2 E_{m}^{\sigma })^{1/2} / \Omega _{ch}$ and $\beta _{h}^{\sigma } = 8 \pi p_{h}^{\sigma } / B_{0}^{2}$. The variables are normalized as *t* → τ_*A*_*t*, $\mathbf {x} \rightarrow a \mathbf {x}$ and $\mathbf {B} \rightarrow B_{0} \mathbf {B}$. The last term in Equation (29) represents the contribution of the perturbed parallel current of CEIs and is proportional to $({\rm d} J_{\parallel ,h0}/{\rm d} r ) \delta \hat{A}_{\parallel }$. This is consistent with the physical picture presented above. Thus, the effects of CEIs depend on their direction of circulation: co-CEIs have a stabilizing role and counter-CEIs a destabilizing one. From Equation (29), without considering a conducting wall, the instability criterion can be derived approximately as [15]

**Figure 2.**
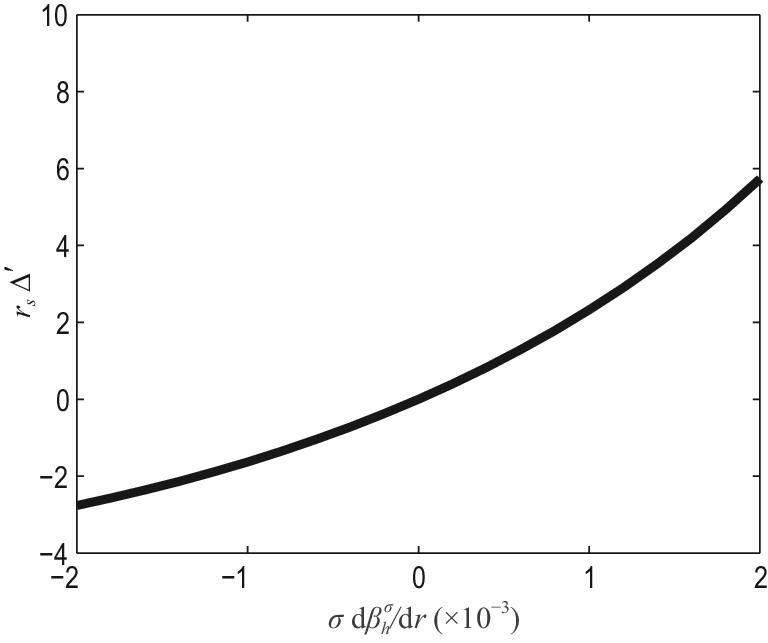
Stability criterion *r_s_*Δ′ of TMs versus the EI pressure gradient σdβ_*h*_/d*r*, where dβ_*h*_/d*r* < 0 and dβ_*h*_/d*r* ∼ −β_*h*_/*r* are assumed, and σ = ±1 for co-circulating and counter-circulating EIs, respectively. Reproduced with permission from [15]. Copyright 2011 American Physical Society.

**Figure 3.**
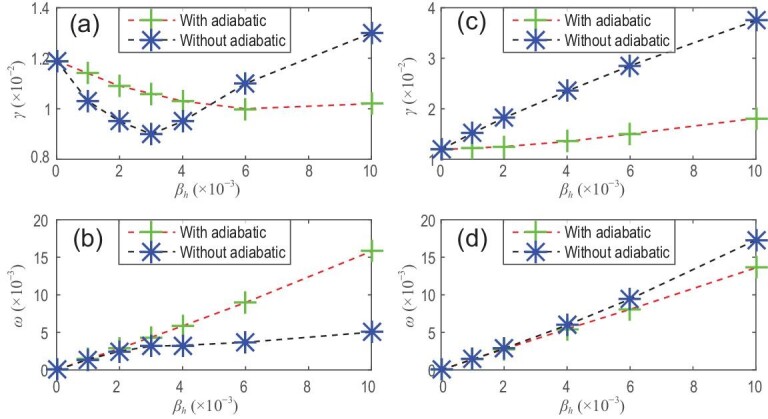
Panels (a) and (b) show the growth rate γ and real frequency ω versus EI β_*h*_ for ρ_*h*_ = 0.07 and *v*_0_ = 0.5 for co-CEIs. Panels (c) and (d) show the growth rate γ and real frequency ω versus EI β_*h*_ for ρ_*h*_ = 0.07 and *v*_0_ = 0.5 for counter-CEIs. Here ρ_*h*_ = *v*_0_/ω_*c*_ with ω_*c*_ the gyrofrequency and *v*_0_ the injection velocity. Reproduced with permission from [16]. Copyright 2012 AIP Publishing.

(30) \begin{eqnarray*} \Delta ^{\prime } = - \frac{\pi \alpha }{r_{s}} \cot \!\big [ \pi \big ( \sqrt{m^{2} + \alpha } - m \big ) \big ], \end{eqnarray*}

where α = α_0_ + α_*h*_, with $\alpha _{h} = \chi _{0} q^{2}(r_{s}) (\varepsilon {\rm d} q(r_{s})/ {\rm d} r )^{-1} \sum _{\sigma } \sigma (\rho _{hm}^{\sigma })^{-1} {\rm d} \beta _{h}^{\sigma } /{\rm d} r$ and α_0_ a critical parameter determining the value of Δ′ in the absence of the effects of EIs as shown in Equation (10). Equation (30) is valid only for 0 < α < 2*m* + 1, since it is derived from the leading-order expansion near the resistive layer of the solution of Equation (29). For the complete solution, it is necessary to solve Equation (29) numerically. A plot of Δ′ against $\sigma d \beta _{h}^{\sigma } / d r$ is shown in Fig. 2. It can be seen that the effects of CEIs on Δ′ are dramatic. For counter-CEIs, Δ′ increases with β_*h*_, and can become positive, i.e. the TM becomes unstable. This can provide the seed islands for NTMs and may be one of the onset mechanisms of spontaneous NTMs. It will also reduce the onset threshold of NTMs. For co-CEIs, Δ′ decreases with β_*h*_, and can become negative or more negative. This will increase the onset threshold of NTMs. The effects of EIs on TMs have also been simulated in [14,16]. In [14], it was shown that the growth rate of TMs is reduced dramatically by EPs, and it can even become zero if the fraction of EIs is large enough, where the effects of CEIs and TEIs were combined, and the background plasma β was large. It should be pointed out that EIs affect TMs mainly through interaction with the outer region. In [16], the effects of CEIs and TEIs were both simulated. In these simulations, the plasma β was assumed to be near zero for simplicity. It can be seen from Fig. 3 that the kinetic effect of co-CEIs is stabilizing, while their adiabatic effect is destabilizing for small β_*h*_. The net effect of both the adiabatic and nonadiabatic contributions is weakly stabilizing. On the other hand, it can be seen that the kinetic effect of counter-CEIs is strongly destabilizing, while the effect from the adiabatic response is stabilizing. The net effect is destabilizing. The results of the simulations of the kinetic effects of CEIs agree in large part with the analytic results presented above. In fact, experiments in DIII-D [8] and ASDEX-U [9] have shown that the onset threshold of NTMs depends on the power of NBI. In the case of the DIII-D experiment, the onset threshold increases with increasing co-NBI power, while remaining almost unchanged as the power of counter-NBI is increased. However, for the ASDEX-U experiment, the onset threshold increases with increasing powers of both co-NBI and counter-NBI. Thus, these two experiments gave different results for counter-NBI. The analytical and simulation approaches presented above cannot yet explain these experimental discrepancies for counter-NBI. However, it is known that the combined effects of EIs and toroidal rotation need to be taken into account, since NBI will induce substantial toroidal rotation, and this will affect MHD instabilities in the following ways.

Unbalanced NBI will supply additional momentum to the plasma and drive a toroidal rotation. Some experiments have revealed Mach numbers up to unity during unbalanced high-power NBI [63]. Much theoretical work has been devoted to understanding the effect of rotation on TMs [21,64–69], and it has been shown that TMs are stabilized mainly by the magnitude of rotation rather than by rotation shear. Physically, toroidal rotation induces centrifugal and Coriolis forces. The equilibrium pressure profile has a poloidal dependence. Perturbations of pressure and density then have sidebands due to compressibility and the poloidal dependence of equilibrium profiles. A perpendicular current is driven by coupling between the magnetic curvature and the pressure, centrifugal and Coriolis forces. Correspondingly, a return parallel current is induced. Thus, the stability of TMs is affected. The analysis in [21] shows that the effect of toroidal rotation is mainly through interaction with the island region, while the effect on Δ′ is small. The stabilizing effect of toroidal rotation on TMs is dramatic and independent of the direction of toroidal rotation. Thus, based on the above results, co-NBI reduces the growth rate of TMs, since CEIs and toroidal rotation both play a stabilizing role. In the case of counter-NBI, whether it is stabilizing or destabilizing depends on which effect dominates among the destabilizing effect of counter-CEIs and the stabilizing effect of toroidal rotation. In the experiment in DIII-D [8], it was found that the onset threshold of NTMs remained almost unchanged when the power of counter-NBI was increased. In this case, the effects of counter-CEIs and toroidal rotation may be balanced. In the experiment in ASDEX-U [9], it was found that the onset threshold of NTMs increased with increasing powers of both co-NBI and counter-NBI. In this case, the effect of toroidal rotation may be dominant. To resolve this issue, self-consistent simulations are required. It is necessary to calculate the dependencies of the driven current and the toroidal rotation on the power of NBI in a consistent manner. These profiles can then be applied in simulations to study the effects of NBI on NTMs. Unfortunately, the appropriate codes have yet to be developed.

### Influence of TEPs in the outer region

In this subsection, we review the interaction between TEPs and TMs in the outer region, which has been studied in [18,19]. Halfmoon and Brennan [18] investigated the effect of TEIs using a reduced model and specific equilibrium profiles. Zhang *et al.* [19] took the finite orbital width into account in their analysis for the effect of TEIs. In contrast to CEIs, the TEI orbits in the (*r*, θ) plane are banana-shaped and concentrated in the low field side. They thus have strong poloidal asymmetry, and their orbits are less likely to be changed by perturbations in comparison with CEIs. More importantly, TEIs do not produce a parallel current directly if the BC is not considered. Thus, the physical picture is different from that of CEIs described above.

However, the methods for analyzing TEIs are similar to those for CEIs. For deeply trapped particles, *r_d_* = *r* − ρ_∥, θ_, ρ_∥, θ_ = *w_b_*cos ω_*b*_*t*, θ = θ_*b*_sin ω_*b*_*t*, α = *q*(*r_d_*)θ − ζ and α = −ω_*d*_*t*, where ω_*b*_ and ω_*d*_ are the bounce and precession frequencies, respectively, of trapped particles, *w_b_* = *v_d_*θ_*b*_/ω_*b*_ is the banana width and θ_*b*_ is the poloidal angle of the turning point. Transforming from coordinates (*r*, θ, ζ) to the particle orbit coordinates (*r_d_*, θ, ζ), by some similar derivation as above, the perturbed distribution of TEIs can be obtained [19]. Then, by applying the integration ∮dθexp (− *im*θ + *in*ζ + *i*ω*t*)/2π to Equation (20), one obtains [19]

(31) \begin{eqnarray*} &&\!\!\!\!\bigg ( \frac{m}{n} - q \bigg ) \frac{1}{q R_{0}} \bigg [\frac{d}{d r} \bigg ( r \frac{d \delta \hat{A}_{\parallel }}{d r} \bigg ) - \frac{m^{2}}{r} \delta \hat{A}_{\parallel } \bigg ] \\ &&\!\!\!\!- \frac{m}{n} \frac{d J_{\parallel ,0}}{d r} \delta \hat{A}_{\parallel }\!+\! \frac{1}{2 q R_{0}} \frac{m^{2}}{n (m \!-\! n q)} \frac{{\rm d} (\beta _{t} \!-\! \beta _{h})}{{\rm d} r} \delta \hat{A}_{\parallel } \\ &&\qquad= 0 , \end{eqnarray*}

where the normalization is the same as that adopted above for CEIs. Here, the parallel current generated by TEIs is ignored. Note that the fluid effect of TEIs is comparable to the kinetic effect, owing to the strong poloidal asymmetry. They tend to cancel each other out. This is reflected in the last term, which is proportional to β_*c*_. Its detailed form can be found in [19]. It can also be seen that the pressure of TEIs is not very sensitive to the perturbations, since it behaves in a stiff manner like a steel wire owing to its high energy. This is also the case for CEIs. Then, from Equation (31), the instability criterion can be found as

(32) \begin{eqnarray*} \Delta _{\beta }^{\prime } &=& \bigg ( \frac{2\,m }{r_{s}} \bigg )^{2 \nu + 1} \frac{1}{2 \nu (1 + 2 \nu )} \frac{\Gamma (1-2 \nu )}{\Gamma (1+2 \nu )}\\ &&\times \, \bigg [ \frac{\Gamma (1- \lambda + \nu )}{\Gamma (-\lambda - \nu )} + \frac{\Gamma (1+ \lambda + \nu )}{\Gamma (\lambda - \nu )} \bigg ],\\ \end{eqnarray*}

where

$$\begin{eqnarray*}
\nu &=& -\frac{1}{2} + \sqrt{D}_{I},\\
D_{I} &=& \frac{1}{4} + \frac{1}{4 \pi } \frac{q^{2} R_{0}}{s^{2}} \beta _{t} \bigg ( \frac{1}{L_{t}} - \frac{\beta _\mathrm{frac}^{h}}{L_{h}}\bigg ), \\
\lambda &=& -\frac{q^{2} R_{0}}{2\,m ({\rm d} q/ {\rm d} r)} \frac{{\rm d} J_{\parallel ,0}}{{\rm d} r},
\end{eqnarray*}$$

**Figure 4.**
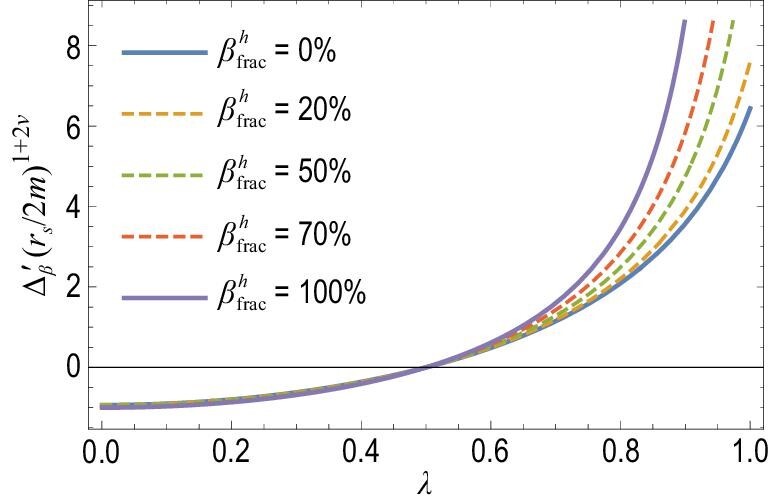
Stability criterion of TMs $\Delta ^{\prime }_{\beta }$ versus λ for different values of $\beta ^{h}_\mathrm{frac}= \beta _{h}/\beta _{t}$, where λ results from the gradient of the equilibrium current density. Note that the threshold condition for these cases is the same as in the zero-beta case. Reproduced with permission from [19]. Copyright 2019 AIP Publishing.

**Figure 5.**
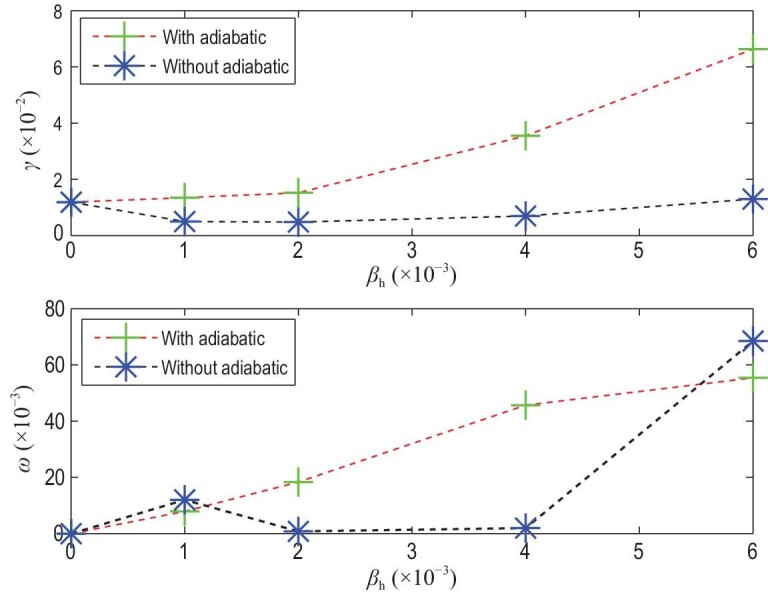
Growth rate γ and real frequency ω versus EI β_*h*_ for ρ_*h*_ = 0.07 and *v*_0_ = 0.5, without/with an adiabatic term for TEI, where ρ_*h*_ = *v*_0_/ω_*c*_, ω_*c*_ is the gyrofrequency, and *v*_0_ is the injection velocity. Reproduced with permission from [16]. Copyright 2012 AIP Publishing.

*D_I_* is the Mercier term at the rational surface, $\beta _\mathrm{frac}^{h} = \beta _{h}/\beta _{t}$, and *L_t, h_* = −dln β_*t, h*_/d*r* are the scale lengths for the pressures of the plasma and the EPs, respectively. It can be seen that the effect of TEIs is proportional to their beta fraction. The main physical basis of the modification comes from the change in the Mercier term due to TEIs. This is shown in Fig. 4, where Δ′ is plotted against λ for different values of $\beta _\mathrm{frac}^{h}$. It can be seen that Δ′ increases with increasing $\beta _\mathrm{frac}^{h}$ for positive Δ′, i.e. TEIs increase the growth rate of TMs and reduce the onset threshold of NTMs. For negative Δ′, the effect of TEIs is small. The nonadiabatic effect of TEIs plays a stabilizing role, while their adiabatic effect is destabilizing. The net effect of TEIs is to increase the growth rate of TMs. These results are consistent with those of the simulation in [16]. This can be seen in Fig. 5, where the effects on TM stability are dominated by the adiabatic part, and the net effect is destabilizing. If β_*h*_ increases beyond a certain threshold, a fishbone-like mode is excited, which will be discussed in the subsection entitled ‘Resonance between EPs and TMs’. In contrast to the effect of CEIs on TMs, the effect of TEIs results from coupling between the bad curvature and the asymmetric pressure of TEIs, since the parallel current generated by TEIs is very small and can be neglected.

### Influence of EPs in the island region

In the above two subsections we discussed the influence of EPs on the TM through their interactions in the outer region. As pointed out above, the particle orbits lie almost completely in the outer region when the island width is much smaller than the width of the particle orbits. In this case, the response of EPs in the island region can be neglected. Based on the quasineutrality condition, an uncompensated current arises from a net $\mathbf {E}\times \mathbf {B}$ current, because the $\mathbf {E}\times \mathbf {B}$ current of EPs is significantly reduced by the effect of orbital averaging in the limit of large orbital width. The $\mathbf {E}\times \mathbf {B}$ current can be expressed as $\mathbf {J}_{\mathbf {E}} = \sum _{\alpha =e,i,h} e_{\alpha } \langle n_{\alpha } \mathbf {v}_{\mathbf {E}}\rangle _{b}$, where $\mathbf {v}_{E} = c \mathbf {E} \times \mathbf {B} / B^{2}$ is the $\mathbf {E}\times \mathbf {B}$ drift. In the absence of EPs and neglecting the effect of the finite width of ion orbits, this current tends to zero. If EPs are present, the effect of averaging over the orbits of these particles tends to be zero in the limit of large orbital width, i.e. $\langle n_{h} \mathbf {v}_{\mathbf {E}}\rangle _{b} \sim 0$, and so $\mathbf {J}_{\mathbf {E}} \sim - e n_{h} \mathbf {v}_{\mathbf {E}}$. Thus, a return parallel current due to this uncompensated current is generated, and this will affect TMs and NTMs. We focus on the effect on the nonlinear evolution of NTMs as discussed in [20] since this effect is small for linear TM. It is shown that the effect is significant when the magnetic shear is weak, and it is stabilizing when the mode frequency is positive in the plasma frame.

Based on the above interpretation, the uncompensated cross-field current $\mathbf {J}_{u} \sim - e n_{h} \mathbf {v}_{E}$. The return parallel current *J*_∥, *u*_ induced by $\mathbf {J}_{u}$ is then determined by

(33) \begin{eqnarray*} \mathbf {B} \cdot \nabla \bigg (\frac{J_{\parallel ,u}}{B} \bigg ) = - \frac{c^2}{4 \pi v_{A} d_{i}} \frac{m}{r} \frac{1}{n_{i0}} \frac{{\rm d} n_{h0}}{{\rm d} r} \frac{\partial \delta \phi }{\partial \xi },\\ \end{eqnarray*}

where *d_i_* = *c*/ω_*pi*_ is the ion inertial length. We can determine δφ from the quasineutrality condition. Then, in the limit of small Larmor radius, from the ion continuity equation and the electron momentum balance equation, δφ can be obtained [20]. Then, based on Equation (33), the return parallel current *J*_∥, *u*_ can then be obtained. Thus, substituting *J*_∥, *u*_ into Equation (5), the contribution of EPs can be included in the island evolution as

(34) \begin{eqnarray*} \frac{8 \pi }{\eta c^{2}} I_{1} \frac{{\rm d} w}{{\rm d} t} = \Delta ^{\prime } + \Delta _{b}^{\prime } + \Delta _{\pi }^{\prime } + \Delta _{u}^{\prime }. \end{eqnarray*}

Here, $\Delta _{u}^{\prime }$ results from the contribution of $\mathbf {J}_{u}$ as

**Figure 6.**
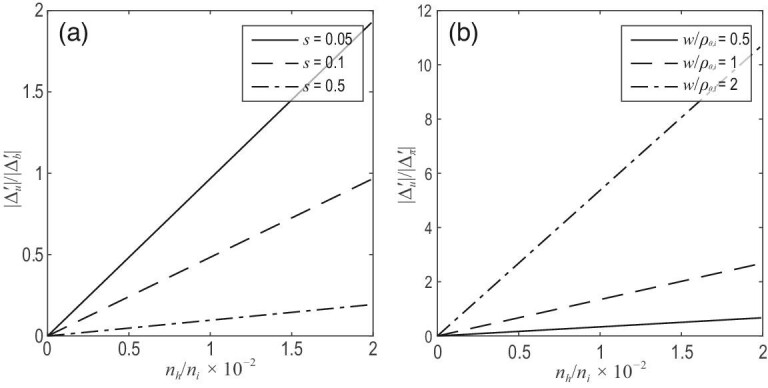
(a) Ratio $|\Delta ^{\prime }_{u}|/|\Delta ^{\prime }_{b}|$ between the contributions of $\mathbf {J}_{u}$ due to EIs and the BC versus the fraction of EI density *n_h_*/*n_i_* for different values of the magnetic shear *s* = 0.05, 0.1 and 0.5. (b) Ratio $|\Delta ^{\prime }_{u}|/|\Delta ^{\prime }_{\pi }|$ between the contributions of $\mathbf {J}_{u}$ due to EIs and the BC versus *n_h_*/*n_i_* for |ω′/ω_*i_ − 1| = 0.5 and *w*/ρ_θ*i*_ = 0.5, 1 and 2. Here ω′ is the island propagation frequency in the plasma frame, ω_*i_ is the ion diamagnetic drift frequency, *w* is the MI width and ρ_θ*i*_ is the ion poloidal Larmor radius. Reproduced with permission from [20]. Copyright 2016 International Atomic Energy Agency.

(35) \begin{eqnarray*} \Delta _{u}^{\prime }=- G_{3} \frac{r_{s}^{2}}{s^{2} L_{n_i}^{2}} \frac{\beta _{\theta i}}{w} \frac{\omega }{\omega _{*i}} \frac{L_{n_{i}}}{L_{h}} \frac{n_{h}}{n_{i}}, \end{eqnarray*}

where the numerical coefficient *G*_3_ ≃ 1.58 and *L_h_* is the scale length of the EI density. Note that Equation (34) is valid for island widths much smaller than the orbital width of the EIs. The seed island to trigger NTMs is always smaller than this orbital width. Equation (34) can then be applied to study the effect of EIs on the onset threshold of NTMs, which is reflected in Δ′ (as discussed above) and $\Delta ^{\prime }_{u}$. From the expression for $\Delta ^{\prime }_{u}$, it can be seen that the effect of $\mathbf {J}_{u}$ depends on the magnetic shear, the island propagation frequency and the EI density gradient at the rational surface. It tends to increase the onset threshold of NTMs for ω < 0 if the density gradients of the thermal ions and the EIs have the same sign at the rational surface. This is different from the effect of the neoclassical polarization current, which is stabilizing for ω > 0 or ω < ω_*i_. The value of $\Delta ^{\prime }_{u}$ is proportional to the ratio *n_h_*/*n_i_*, which is small, but can be up to $O(1\%)$ in ITER. For a typical tokamak like JT-60U, the EI density can be up to $2\% \times n_{i}$ during NBI [70]. Although the ratio *n_h_*/*n_i_* is small, $\Delta ^{\prime }_{u}$ may become significant for weak magnetic shear, as in steady-state and hybrid operational scenarios in ITER and in some large tokamaks [7], where many steady operational discharges have been realized with a configuration of zero or weak magnetic shear. For weak magnetic shear, the effect of $\mathbf {J}_{u}$ can be comparable to the contribution of the BC, and will increase the onset threshold of NTMs or suppress them. This is shown in Fig. 6(a), where $|\Delta ^{\prime }_{u}/\Delta ^{\prime }_{b}|$ is plotted against *n_h_*/*n_i_* for different values of the magnetic shear. It can be seen that $|\Delta ^{\prime }_{u}/\Delta ^{\prime }_{b}|$ increases as *n_h_*/*n_i_* increases or *s* decreases. For weak magnetic shear and a large fraction of EI density, $|\Delta ^{\prime }_{u}/\Delta ^{\prime }_{b}| \sim 1$ or >1, i.e. the contribution of $\mathbf {J}_{u}$ becomes dramatic, and its stabilizing effect can partially cancel or overcome the destabilizing effect of the BC. Thus, the onset threshold of NTMs is increased, and NTMs can even be suppressed. To provide a comparison between $\Delta _{u}^{\prime }$ and $\Delta ^{\prime }_{\pi }$, because they have the same dependence on *s* but different dependencies on *w*/ρ_θ, *i*_, Fig. 6(b) presents a plot of the ratio $|\Delta ^{\prime }_{u}/\Delta ^{\prime }_{\pi }|$ against *n_h_*/*n_i_* for different values of *w*/ρ_θ, *i*_. It can be seen that $|\Delta ^{\prime }_{u}/\Delta ^{\prime }_{\pi }| \sim 1$ for *w*/ρ_θ, *i*_ ∼ 1 and $n_{h}/n_{i} \sim 1\%$, which are typical values in a tokamak. Owing to the different dependencies on ω, the effects are opposite for ω_*_ < ω < 0. In this case, the neoclassical polarization current is destabilizing, while the uncompensated cross-field current is stabilizing, and they tend to cancel each other out. Thus, the onset threshold is increased by the neoclassical polarization current. In some experiments in tokamaks, like those in JT-60U [70], no NTMs were observed in discharges during NBI with weak magnetic shear and a small pressure gradient at the resonance surface, where the effects of EIs may be important.

**Figure 7.**
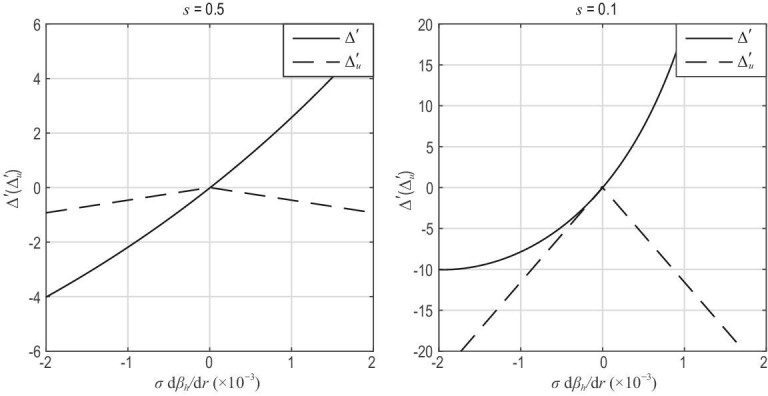
Plots of $\Delta ^{\prime }_{u}$ and the stability criterion of TMs Δ′ including the contribution of EIs versus σdβ_*h*_/d*r*, where β_*h*_ = 8π*p_h_*/^2^, σ = +1 for co-CEIs, σ = −1 for counter-CEIs and *p_h_* is the pressure of EIs. Reproduced with permission from [20]. Copyright 2016 International Atomic Energy Agency.

As reviewed above, the effects of EIs are reflected in Δ′ owing to the interaction of EIs with the outer region and in $\Delta ^{\prime }_{u}$ owing to the interaction with the island region, where a contribution from the uncompensated cross-field current is generated owing to quasineutrality. These two effects are shown in Fig. 7, where it can be seen that they both increase with decreasing magnetic shear. At sufficiently small magnetic shear, $\Delta ^{\prime }_{u}$ becomes comparable to Δ′ and they both become more significant. In ITER this provides the possibility of using EIs to enhance the onset threshold or suppress NTMs for the steady state and hybrid scenarios with weak magnetic shear. Note that Δ′ depends on the direction of circulation. By combining these two effects, it might be possible to increase the onset threshold of NTMs or suppress them using co-CEIs by optimization of the *q* profile, where the weak magnetic shear is beneficial in enhancing this suppressive effect.

### Influence of CEPs in the island region

In this subsection, we review the influence of CEIs on NTMs in the island region based on [13] and make some comments. An interpretive picture is given first. It is known that the orbits of CEIs drift from the magnetic surface due to magnetic drift. Whether this drift is outward or inward depends on the direction of circulation *v*_∥_. As shown in [13], the equilibrium magnetic field near the rational surface in a slab geometry can be written as $\mathbf {B} = B_{0} \mathbf {e}_{z} + (B_{y} x/L_{s}) \mathbf {e}_{y}$, where *L_s_* is the local shear length. If $\delta \mathbf {B} = b_{0} \sin {k y} \mathbf {e}_{x}$ is imposed, an MI forms near the rational surface. The guiding center motion of EPs in the equilibrium magnetic field is $\mathbf {v}_{0} = v_{\parallel } \mathbf {e}_{z} + v_{\parallel } B_{y}/(B_{0} L_{s}) (x-x_{*}) \mathbf {e}_{y}$, $x_{*} = -(v_{\parallel } + v_{\perp }^{2}/2 v_{\parallel }) L_{s} B_{0}/(\omega _{c} L_{B} B_{y})$, *L_B_* = (dln *B*/ln *x*)^−1^. The drift motion shifts the null line of *v_y_* with respect to that of *B_y_* by a distance x_*_ from *x* = 0. It can be known that in the presence of an MI, the orbit of CEIs displays a drift island structure shifted by a distance x_*_ from the MI. It should be pointed out here that x_*_ depends on the poloidal angle in a toroidal geometry, even for CEIs, since *L_B_* depends on the poloidal angle. For CEIs, a considerable electric current can be produced. The drift island structure causes an asymmetry of the driven current with respect to the MI. Perturbations of this asymmetry current will affect TMs. The details are given next. The drift kinetic equation of EIs is

(36) \begin{eqnarray*} &&\frac{v_{\parallel }}{q\! R} \bigg [ \frac{\partial }{\partial \theta } + m \bigg (1 - \frac{q}{q_{s}} \bigg ) \frac{\partial }{\partial \xi } - m \frac{\partial \delta \psi }{\partial \xi } \frac{\partial }{\partial \psi } \bigg ] f_{h} \\ &&\quad+\,\frac{v_{\parallel }}{q\! R} \bigg [ \frac{\partial }{\partial \theta } \bigg ( \frac{v_{\parallel } I}{\omega _{c}} \bigg ) \frac{\partial }{\partial \psi } - \frac{\partial }{\partial \psi }\bigg ( \frac{v_{\parallel } I}{\omega _{c}} \bigg )\\ &&\quad\times \, \bigg ( \frac{\partial }{\partial \theta } + m \frac{\partial }{\partial \xi } \bigg ) \bigg ]f_{h} \\ &&\qquad = C(f_{h}) + S, \end{eqnarray*}

where the explicit time dependence is neglected, since the growth rate of the MI is much smaller than the transit frequency of EIs. The electrostatic potential is also neglected, since *e*δφ/*E_h_* ≪ 1, where *E_h_* is the energy of the EIs. For convenience, the coordinates (ψ, θ, ξ) are chosen here, which is different from the choice of coordinates (ψ, ζ, ξ) in [13]. The quantity δ = ν_*e*_/ω_*te*_ is used as a small parameter, where ν_*e*_ is the electron–ion collision frequency, ω_*te*_ = *v_te_*/*qR* is the electron transit frequency, $v_{te} = \sqrt{2 T_{e}/m_{e}}$ is the electron thermal velocity and *T_e_* is the electron temperature. The ordering is taken as *w*/*a* ∼ δ^2^, *n_h_*/*n_e_* ∼ δ^2^ and *T_e_*/*E_h_* ∼ δ^2^, where *w* is the MI width. Then, β_*h*_ ∼ β_*c*_, where β_*h, c*_ represent the β of the EIs and the core plasma, respectively. Thus, the terms in Equation (36) are of relative order 1: δ^2^: δ^2^: δ^2^*w_b_*/*w*: *w_b_*/*w*: δ^4^: δ^4^, where *w_b_* = *q*ρ_*h*_ is the drift orbital width of CEIs, with ρ_*h*_ = *v*_∥_/ω_*c*_. Based on the above ordering, if *w_b_* ∼ *w* (i.e. the drift orbital width of CEI is comparable to the MI width) then the orbits of CEIs will partially cross the MI, and the physics will become complex. It then becomes difficult to deal with Equation (36) analytically. Assuming that *w_b_* ≪ *w* (i.e. that the orbits of CEIs are fully inside the MI), Equation (36) can be expanded as

(37) \begin{eqnarray*} \frac{\partial f_{h0}}{\partial \theta } = 0, \end{eqnarray*}

(38) \begin{eqnarray*} &&\frac{v_{\parallel }}{q\! R} \frac{\partial f_{h1}}{\partial \theta } + \frac{v_{\parallel }}{q\! R} \bigg [ m \bigg (1 - \frac{q}{q_{s}} \bigg ) \frac{\partial }{\partial \xi }\\ &&\quad -\, m \frac{\partial \delta \psi }{\partial \xi } \frac{\partial }{\partial \psi } \bigg ] f_{h0} \\ &&\quad+\,\frac{v_{\parallel }}{q\! R} \bigg [ \frac{\partial }{\partial \theta } \bigg ( \frac{v_{\parallel } I}{\omega _{c}} \bigg ) \frac{\partial }{\partial \psi }\\ &&\quad -\, \frac{\partial }{\partial \psi }\bigg ( \frac{v_{\parallel } I}{\omega _{c}} \bigg ) m \frac{\partial }{\partial \xi } \bigg ]f_{h0} \\ &&\qquad = 0, \end{eqnarray*}

(39) \begin{eqnarray*} &&\frac{v_{\parallel }}{q\! R} \frac{\partial f_{h2}}{\partial \theta }+ \frac{v_{\parallel }}{q\! R} \bigg [ m \bigg (1 - \frac{q}{q_{s}} \bigg ) \frac{\partial }{\partial \xi }\\ &&\quad -\, m \frac{\partial \delta \psi }{\partial \xi } \frac{\partial }{\partial \psi } \bigg ] f_{h1} \\ &&\quad+\frac{v_{\parallel }}{q\! R} \bigg [ \frac{\partial }{\partial \theta } \bigg ( \frac{v_{\parallel } I}{\omega _{c}} \bigg ) \frac{\partial }{\partial \psi } - \frac{\partial }{\partial \psi }\bigg ( \frac{v_{\parallel } I}{\omega _{c}} \bigg )\\ &&\quad\times \, \bigg ( \frac{\partial }{\partial \theta } + m \frac{\partial }{\partial \xi } \bigg ) \bigg ]f_{h1} \\ &&\qquad = C(f_{h0}) + S. \end{eqnarray*}

From Equation (37), *f*_*h*0_ = *f*_*h*0_(ψ, ξ, *E*, λ). Then, taking the orbital average ∮(·)*qR*/*v*_∥_ of Equation (39), it is found that

(40) \begin{eqnarray*} \bigg [ m \bigg (1 &-& \frac{q}{q_{s}} \bigg ) \frac{\partial }{\partial \xi } - m \frac{\partial \delta \psi }{\partial \xi } \frac{\partial }{\partial \psi } \bigg ] f_{h0}\\ && +\, n \bar{\omega }_{d} \frac{\partial f_{h0}}{\partial \xi }= 0, \end{eqnarray*}

where $\bar{\omega }_{d} = - \oint ({\rm d} \theta /2 \pi )\, \partial (v_{\parallel } I / \omega _{c})/\partial \psi$. Equation (40) can be rewritten as

(41) \begin{eqnarray*} \bigg [ \frac{q_{s}^{\prime }}{q_{s}} (x- x_{*}) \frac{\partial }{\partial \xi } + \frac{\partial \delta \psi }{\partial \xi } \frac{\partial }{\partial \psi } \bigg ]f_{h0} =0, \end{eqnarray*}

where $x_{*} = \bar{\omega }_{d} / q_{s}^{\prime }$ is the net drift distance given in [13]. It can be shown that x_*_ ∼ (*q_s_*ρ_*h*_/2*s*)*D*, with *D* = 2ε(1/*q*^2^ − 1) − 3Λ′ − *r*Λ″ − ε*s* [71], where Λ is the Shafranov shift. It then follows that x_*_/*w_b_* ∼ ε, i.e. the net drift distance is much smaller than the drift orbital width. Thus, x_*_ ≪ *w*, based on the above ordering. However, the assumption that x_*_ ≥ *w* is made in [13].

Introducing the drift island coordinates (Ω_*_, ξ_*_), with Ω_*_ = (2/*w*^2^)(*x* − x_*_)^2^ − cos ξ_*_ and ξ_*_ = ξ, Equation (41) can be written as

(42) \begin{eqnarray*} \frac{q_{s}^{\prime }}{q_{s}} \left(x- x_{*}\right) \frac{\partial f_{h0}}{\partial \xi _{*}} =0. \end{eqnarray*}

Thus, it is found that

(43) \begin{eqnarray*} f_{h0}= f_{h0}(\Omega _{*},E,\lambda ). \end{eqnarray*}

This is the same as Equation (27) of [13]. In [13], the subsidiary ordering *w*/x_*_ ≪ 1 is imposed, so that near *x* = 0 one obtains

(44) \begin{eqnarray*} f_{h0} \sim f_{h0}(x=0) - \frac{\partial f_{h0}}{\partial (x-x_{*})} \frac{q_{s} \delta \hat{\psi } \cos {\xi }}{q_{s}^{\prime }},\\ \end{eqnarray*}

which is the same as Equation (32) of [13]. However, the results obtained in [13] are not consistent, since the net drift x_*_ is overestimated, giving a subsidiary ordering *w*/x_*_ ≪ 1 that is contrary to the previous ordering x_*_ ≪ *w*. In fact, one can understand this as follows. If x_*_ ≥ *w* then the drift orbital width *w_b_* ≫ *w*, since x_*_/*w_b_* ∼ ε. Now, the orbits of the CEIs are almost completely outside the MI, since the orbital width is much larger than the MI width. Thus, the effect of EIs inside the MI is expected to be small, because the response of the EIs to perturbations in the island region is weakened by orbital averaging [20]. The trajectories of the EIs are almost insensitive to the MI, since its width is much smaller than their orbital width. In the case of an MI width much smaller than the orbital width, the results of the above three subsections apply.

To investigate the influence of EPs on TMs, it is assumed that the MI width is much smaller than the orbital width. This assumption is always satisfied for the linear phase, the early nonlinear phase and the onset threshold of NTMs. As the MI increases, its width will become comparable to or larger than the orbital width. Then, the EP physics will be different, since the picture in which the orbits are almost completely in the outer region is no longer valid. In an experiment in DIII-D [12], it was found that the effect of EPs is weak for MI widths much larger than the orbital width. To help explain this experimental result, a simulation was performed using a global gyrokinetic toroidal code (GTC) in which a perturbed parallel current induced by EPs was added to Ampére’s law [23]. According to this simulation, EPs have a weakly stabilizing effect on NTMs. Only the perturbed parallel current was included in the code, and the effect of EPs on equilibrium was not considered. The effects of EPs and toroidal rotation on equilibrium and instabilities have been shown to be important [72]. In fact, up to now, there are still no self-consistent codes for describing NTMs in which both the physics of the EPs and the effects of toroidal rotation are taken into account. The development of such codes is essential if a deeper understanding of the physics of NTMs is to be obtained.

### Resonance between EPs and TMs

**Figure 8.**
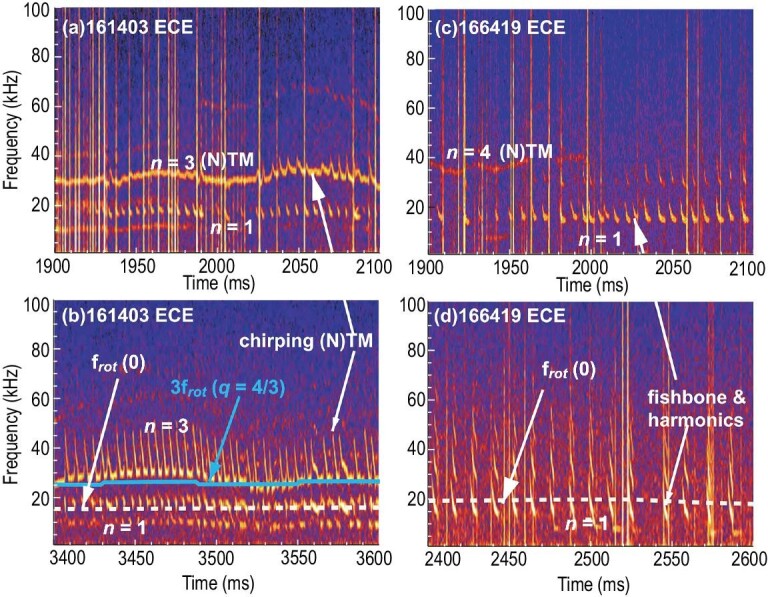
Mode frequencies with time (measured by the electron cyclotron emission diagnostic) for two hybrid discharges in DIII-D: (a) shot 161403 from 1900 to 2100 ms; (b) shot 161403 from 3400 to 3600 ms; (c) shot 166419 from 1900 to 2100 ms; (d) shot 166419 from 2400 to 2600 ms. In (a) and (b), steady (N)TMs and chirping *n* = 1 fishbones coexist for more than 100 ms and then chirping (N)TMs and chirping fishbones coexist. In (c), chirping (N)TMs and chirping fishbones coexist for more than 200 ms. In (d), (N)TMs are fully stabilized and fishbones are dominant. The toroidal rotation frequency at the central and the *q* = 4/3 rational surface are plotted with dashed white and blue lines. Reproduced with permission from [31]. Copyright 2020 International Atomic Energy Agency.

**Figure 9.**
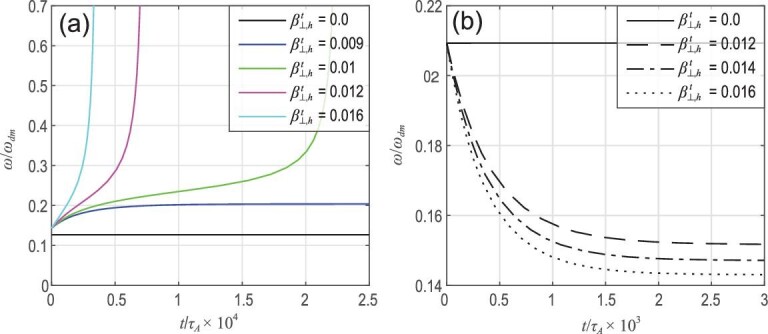
Frequency against time for different EIs β_*h*_: (a) island propagates in the ion diamagnetic drift direction with magnetic shear *s* = 0.5; (b) island propagates in the electron diamagnetic drift direction with magnetic shear *s* = −0.5. Reproduced with permission from [33]. Copyright 2021 International Atomic Energy Agency.

Generally, the frequency of TMs is much smaller than the precession and bounce/transit frequencies of EPs, and resonance between EPs and TMs rarely occurs. However, a fast frequency chirping during TMs has been observed in some tokamaks [27–31]. During such frequency chirping, a measurable reduction in the neutron rate is found. This indicates that a strong resonance occurs in this process. The frequency first jumps up from the TM frequency to a high value, and then chirps back down to the TM frequency. This happens within ∼1 ms. This phenomena can be seen in the experimental results from DIII-D shown in Fig. 8 [31]. The chirping phenomena has been understood from two aspects: one understanding is that it is a new mode, a fishbone-like mode [34,35]; one understanding is that it is still NTM but it is a so-called chirping NTM [32,33]. Marchenko and Lutsenko [32] tried to explain this by resonance between TEPs and NTM, providing an additional toroidal torque to accelerate rotation of the MI in the direction of the ion diamagnetic drift. They calculated the evolutions of the MI and the rotation frequency, taking account of this resonance effect. However, the calculated time over which the frequency chirps up and down is much larger than 1 ms (by a factor of 10^2^). In recent work, in contrast to the particle model used in [32], the physical basis of the DIII-D experimental results is re-examined by drift kinetic theory self-consistently. The resonance between trapped EIs and NTM significantly affect the evolution of NTM frequency, which can be seen in Fig. 9. For the island propagating in the ion diamagnetic drift direction, NTM frequency chirps up when the EIs β_*h*_ exceed a critical value. For the island propagating in the electron diamagnetic drift direction, the frequency chirping does not occur. The calculated chirping time and predicted island propagation direction are consistent with DIII-D experimental results [31]. An experiment in HL-2A has shown that a fishbone-like mode may arise during the frequency chirping associated with TMs. Attempts have been made to explain this both analytically [35] and through simulations [34]. In fact, the basic physics of the fishbone-like mode is similar to that for a fishbone mode, with resonance inducing an energetic-particle-driven mode. This can be analyzed based on the generalized energy principle [35]. In the analysis by Zhang *et al.* [35], it was shown that a fishbone-like mode is excited by resonance between the TM and EPs, and that the resonance between CEPs and the TM can be neglected in the limit of small orbital width. When β_*h*_ exceeds a certain threshold, a fishbone-like mode is excited. Then, a TM and a fishbone-like mode coexist. As β_*h*_ increases, the fishbone-like mode becomes dominant. A similar phenomenon was also found in [73], where the interaction between a double TM and TEPs was simulated. It was shown that the double TM and a fishbone-like mode could coexist for a certain value of β_*h*_. At higher values of β_*h*_, only the fishbone-like mode could be found in the simulation. In the analytical work by Zhang *et al.* [35], it was claimed that resonance between CEPs and TMs in the limit of small orbital width can be neglected. However, the simulation by Zhu *et al.* [34] showed that a fishbone-like mode was excited by resonance between co-CEPs and the TM. In this simulation, the effects of a finite orbital width were taken into account, while the background plasma beta was set to be nearly zero. Owing to a lack of experimental data on the distribution of EPs, it is hard to judge which mechanism is applicable, and further investigations are needed.

It can be seen from the above experimental and theoretical results that it remains an open question as to whether the frequency chirping arises from a so-called chirping NTM/TM or from an energetic-particle-driven mode. More detailed experimental measurements and theoretical studies are needed to clarify the mechanism.

Based on the discussions in the above subsections, the effects of EIs and rotation on TMs in the limit in which the orbital width of EIs is much larger than the MI width are summarized in Table 1.

**Table 1.** Effects of EIs and rotation on TMs.

	Effects in the outer region	Effects in the resistive layer
Rotation	Negligible	Stabilizing
Co-passing ions	Stabilizing	Stabilizing if $\omega \frac{{\rm d}n_{h}}{{\rm d} r} \frac{{\rm d}n_{i}}{{\rm d} r} > 0$
Counter-passing ions	Destabilizing	
Trapped ions	Destabilizing	

## INFLUENCE OF TMs ON EP TRANSPORT

### Introduction

The issue of EP transport is important for MCF plasmas. For confinement of EPs, the neoclassical transport of EIs is much smaller than neoclassical transport of thermal ions, owing to the low collision frequency of the EIs. Anomalous transport of EIs due to turbulence is also much smaller than that of thermal ions, owing to gyroradius averaging and orbital averaging. In addition to the above, there are some loss channels.

1. Prompt loss: after the production of EPs, some EPs will strike the first wall before completing their orbits.
2. Ripple loss: toroidal field ripples due to the discreteness of the toroidal field coils will cause toroidal asymmetry, which will have a dramatic effect, mainly on trapped or barely trapped particles, and can cause considerable loss of EPs.
3. Loss due to plasma instabilities: plasma instabilities will introduce electric and magnetic field perturbations, which can distort particle orbits and cause changes in the constants of motion. These will lead to loss and redistribution of EPs. One can classify these losses as resonant or nonresonant. For resonant losses, an important transport mechanism is convective transport of EPs in phase space, as shown in [74]. This can lead to radial drift of EPs due to changes in the constants of motion during frequency chirping [75].

In this review, we focus on the transport of EPs by MIs generated by TMs (including neoclassical TMs). MIs can have a significant and negative impact on plasma confinement, and they can also lead to loss and redistribution of EPs. Since the orbits of circulating particles and trapped particles are different, their transport mechanisms in the presence of MIs are different, and we review them separately.

### Influence of TMs on the transport of CEPs

For CEPs, the resonance between EPs and TMs is hardly satisfied, due to the mode frequency being much smaller than the transit frequency of EPs. The main loss mechanism of CEPs is caused by the formation of drift islands. The following is a heuristic physical picture. The circulating particles follow the magnetic field lines. If a particle is of low enough energy, its orbit will be almost identical to a magnetic field line. In fact, the contours of the magnetic field can be plotted by the orbits of circulating electrons with very low energy. Then, if the magnetic field topology acquires an MI structure, it can be assumed that the orbits of circulating particles have a similar island structure. Owing to the magnetic drift of EPs, the orbits form a so-called drift island, the width of which is proportional to that of the MI. The magnetic drift depends on the poloidal angle: it is proportional to cos θ and sin θ. This behavior can be thought of as being due to the addition of an *n* = 0, *m* = ±1 perturbation to the particle orbits. When one plots the Poincáre map of the orbits, *n, m* ± 1 islands will appear, associated with beating between the TM and magnetic drift. These drift island structures will strongly affect EP transport. If the width of the drift islands is larger than a certain threshold determined by the MI width or the particle energy, drift islands will overlap, and trigger stochasticity. This will lead to a dramatic loss of CEPs. This is the main loss mechanism for CEPs, and we now review it based on Mynick’s work [37].

A single helical radial magnetic perturbation of the TM is given as δ*B_r_* ∼ *B*_0_*b*(*r*)cos ξ, where ξ = *m*θ − *n*ζ + φ_*nm*_ is the helical angle and φ_*mn*_ = φ_*mn*0_ − ω*t* is an arbitrary mode phase, which can be assumed to be a constant since the transit frequency of EPs is much larger than the TM frequency. One has

(45) \begin{eqnarray*} \frac{{\rm d} r_{d}}{{\rm d} t} = v_{\parallel } b(r) \cos {\xi }, \end{eqnarray*}

where $\mathbf {E} \times \mathbf {B}$ drift is ignored. Details about the orbit due to perturbations can be found in [76]. Without perturbation, the orbit of an EP that is not a barely passing/trapped particle is *r* ≃ *r_d_* + *r*_1_cos θ_*b*_, θ ≃ τθ_*b*_ + θ_1_sin θ_*b*_, ζ ≃ *q*(*r_d_*)θ_*b*_ + ζ_1_sin θ_*b*_, *v*_∥_ = τ*u*_0_ + *u*_1_cos θ_*b*_, where *r_d_* is the particle’s bounce average radius, θ_*b*_ is the bounce phase, and τ = 1 for circulating particles and 0 for trapped particles. Here, θ and ζ are separated into secular and oscillating parts. The factor cos θ_*b*_ = ∑_*l*_*J_l_*(ξ_1_)cos ξ_*l*_, where *J_l_* are Bessel functions, ξ_*l*_ = (*m* − *nq* + *l*)θ_*b*_ + φ_*mn*_ and ξ_1_ = *m*θ_1_ − *n*ζ_1_ is one-half the change in mode phase during a transit time due to the oscillatory portion of the motion resulting from magnetic drift. The mode amplitude *b*(*r*) ≃ *b*_0_ + (*r* − *r_d_*)d*b*/d*r* ≡ *b*_0_ + *b*_1_cos θ_*b*_, where it is assumed that the mode amplitude changes little over the scale of the particle banana width. Equation (45) can then be written as

(46) \begin{eqnarray*} \frac{{\rm d} r_{d}}{{\rm d} t} = \sum _{l} v_{l} \cos {\xi _{l}}, \end{eqnarray*}

where

(47) \begin{eqnarray*} v_{l} &=& \left ( u_{0} b_{0} + \frac{1}{2}u_{1} b_{1} \right ) J_{l} + \frac{1}{2} ( u_{0} b_{1} + b_{0} u_{1} )\\ &&\times \, (J_{l-1} + J_{l+1} ) + \frac{1}{4} u_{1} b_{1} ( J_{l-2} + J_{l+2} ).\\ \end{eqnarray*}

From Equation (46), it can be seen that the main drift island *m* comes from the *l* = 0 contribution, while the additional *m* ± 1 comes from the *l*∓1 contributions. If *u*_1_ ≪ 1 and *b*_1_ ≪ 1, only the *l* = 0 contribution survives, and the drift island becomes nearly driftless because *u*_1_ ≪ 1. This can be seen in Fig. 4 of [37]. To estimate the width of the drift island, the time development of the phase ξ_*l*_ is needed. One has

(48) \begin{eqnarray*} \Omega _{l} \equiv \frac{{\rm d} \xi _{l}}{{\rm d} t} = (m + l - n q(r_{d}) ) \omega _{b} - \omega . \end{eqnarray*}

Similar to dealing with the MI geometry, by expanding Equation (48) near the rational surface *q*_*m*+*l*_ where Ω_*l*_ = 0, the contour of the *m* + *l* drift island can be expressed as $\Theta _{l} = \Omega _{l}^{\prime }/2 ( r_{d} - r_{l} )^{2} - v_{l} \cos {\xi _{l}}$. Then, the half-width of the *m* + *l* drift island at the *m* + *l*/*n* rational surface can be obtained as $\delta r_{m+l} = ( 4 v_{l}/\Omega _{l}^{\prime } )^{1/2}$. Thus, the condition for overlap of adjacent drift islands is

(49) \begin{eqnarray*} \delta r_{m+l+1} + \delta r_{m+l} \ge r_{m+l+1} - r_{m+l}, \end{eqnarray*}

where *r*_*m*+*l*_ = *r*(*q*_*m*+*l*_) is the location of the rational surface *q*_*m* + *l*_. Equation (49) is also the stochastic threshold. For a given equilibrium profile, this threshold depends on the TM amplitude and the particle energy. If these are sufficiently large then adjacent drift islands *m* and *m* + 1 overlap and lead to stochasticity, then a dramatic loss of CEPs. This mechanism has been confirmed experimentally [12,36,39,40] and in simulations [41,46]. In an experiment in TFTR, it was found that in the presence of a large MI, the loss of alpha particles was increased by up to a factor of five compared with the first orbital loss level [36], which has been explained by the above mechanism in [37]. In an experiment in ASDEX-U, it was shown that the loss of EIs results mainly from the drift island formed by circulating EIs in phase space. Overlap of drift islands leads to orbital stochasticity and increases the loss of EIs to the same order as their prompt loss [40]. The TRANSP ‘Kick’ EP transport model was extended to study NTM-driven EI transport and the results were compared with those from a DIII-D experiment [41], which confirms the drift island mechanism. It was found that, when the MI is smaller than a certain threshold, EI transport is dominated by energy and momentum redistribution. For example, an *m*/*n* = 3/2 NTM leads to a hollow profile of neutral beam torque and a broadened profile of NBI-driven current in the core. When the MI is greater than the threshold, the loss of EIs is increased, and the NBI-driven torque and current decrease across the entire plasma. The threshold is determined by the condition for overlap of drift islands.

### Influence of TMs on the transport of TEPs

In contrast to the orbits of CEPs, those of TEPs do not exhibit a drift island structure. However, the loss of TEPs in the presence of an MI is again significant, as has been found and explored in both experimental and theoretical studies [12,41,43,44,46]. The resonance condition for trapped particles can be written as *l*ω_*b*_ − *n*ω_*d*_ − ω = 0, where ω_*b*_ and ω_*d*_ are the bounce and precessional frequencies, respectively. For TMs, ω ≪ ω_*d*_ and ω ≪ ω_*b*_. Resonance between TEPs and TMs does not always occur. In the absence of such resonance, an MI plays a similar role to a ripple field in introducing a toroidal asymmetry. Magnetic perturbation leads to a small vertical displacement near the banana turning points during every bounce period. This radial shift of the turning points will cause loss of EPs. However, the magnetic perturbation of the TM is much smaller than that caused by a ripple field, and so this loss of TEPs due to the TM is much smaller than ripple-field-induced loss. Here, we would like to review the other mechanism, namely, loss due to resonance between TEPs and TMs. Since ω_*b*_ ∝ *E*^1/2^ and ω_*d*_ ∝ *E*, these will match when *E* increases sufficiently. The resonance condition can then be satisfied, even in the limit of vanishing mode frequency. In this case, magnetic perturbation will give rise to radial displacement of TEPs and lead to their loss. Next, we review this resonant loss mechanism based on the simulation by Poli *et al.* [44]. The time evolutions of the radial displacement and *v*_∥_ are

(50) \begin{eqnarray*} \dot{r} = v_{d,r} + v_{E,r} + v_{r}^{\tilde{B}}, \end{eqnarray*}

(51) \begin{eqnarray*} \dot{v}_{\parallel } \simeq - \mu \mathbf {b}_{0} \cdot \nabla B + \dot{v}_{\parallel }^{\mathrm{mirror},\tilde{B}}, \end{eqnarray*}

where $v_{r}^{\tilde{B}} = v_{\parallel } b(r) \cos {\xi }$, $\dot{v}_{\parallel }^{\mathrm{mirror},\tilde{B}} = - \mu b(r) \cos {\xi }\, \partial B/\partial r$ are the island-related terms. The mirror force due to magnetic perturbation will lead to a shift of the bounce turning points with respect to the unperturbed case. Here cos ξ = ∑_*l*_*J_l_*(ξ_1_)cos ξ_*l*_, where ξ_*l*_ = (*l*ω_*b*_ − *n*ω_*d*_ − ω)*t* + φ_*mn*0_ and ξ_1_ = (*m* − *nq*)θ_1_. It is evolved in the unperturbed orbit. Take the orbital average

(52) \begin{eqnarray*} \left\langle v_{r}^{\tilde{B}} \right\rangle _{b} = \bigg \langle \sum _{l} v_{l,t} (\xi _{1}) \cos {\xi _{l}}\bigg \rangle _{b}, \end{eqnarray*}

where

(53) \begin{eqnarray*} v_{l,t} &=& \frac{1}{2}u_{1} b_{1} J_{l} + \frac{1}{2}b_{0} u_{1} (J_{l-1} + J_{l+1} )\\ && +\, \frac{1}{4} u_{1} b_{1} ( J_{l-2} + J_{l+2} ), \end{eqnarray*}

**Figure 10.**
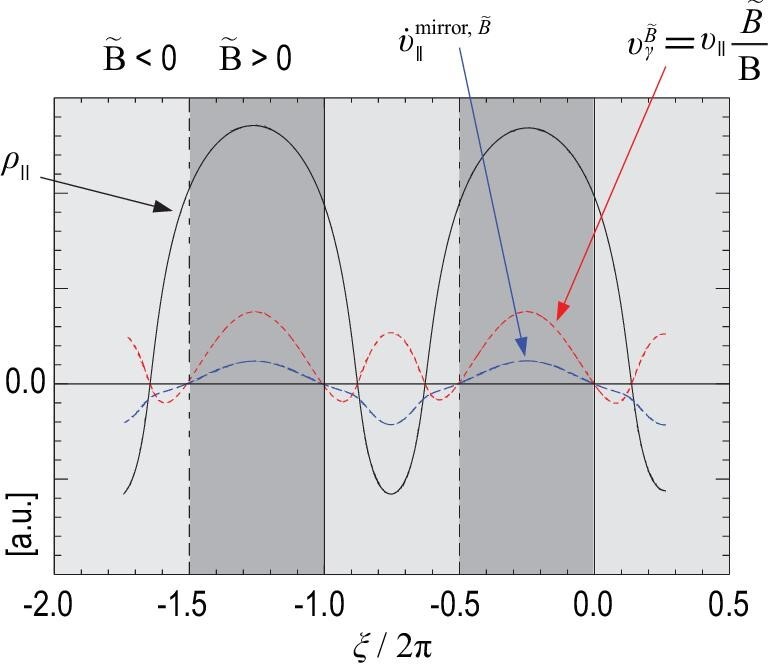
Motion of a trapped particle satisfying the condition ω_*b*_ = *n*ω_*d*_ against ξ/(2π). The solid line represents ρ_∥_, whereas the dotted and dashed lines represent $v_{r}^{\tilde{B}}$ and $\dot{v}_{\parallel }^{\mathrm{mirror},\tilde{B}}$, respectively (both multiplied by an arbitrary factor to fit on the scale). Here ω_*b*_ and ω_*d*_ are the bounce and precessional frequencies of trapped particles, respectively, ξ = *m*θ − *n*ζ + φ_*nm*_ is the helical angle and ρ_∥_ = *v*_∥_/*B*. Reproduced with permission from [44]. Copyright 2008 AIP Publishing.

τ_*b*_ = 2π/ω_*b*_ is the bounce period of trapped particles and $\langle \cdot \rangle _{b}= \int _{t}^{t+\tau _{b}} (\cdot )\, {\rm d} t/\tau _{b}$. Then, for thermal particles with ω ≪ ω_*d*_ ≪ ω_*b*_, the perturbation field that the particles experience is nearly constant during a single bounce period, since ξ is almost constant. However, *v*_∥_ changes direction during each bounce period, and so the bounce average of the radial displacement is nearly zero. This can be seen explicitly using Equation (52), from which $\langle v_{r}^{\tilde{B}} \rangle _{b} \sim 0$ can be obtained. In the case of EPs, the precessional frequency is large, and so the perturbation field felt by EPs during a single bounce period is no longer constant. It can be found from Equation (52) that the perturbation field will change significantly if ω_*d*_ → ω_*b*_. The bounce average of the radial displacement reaches its maximum value if the resonance condition ω_*b*_ − ω_*d*_ − ω = 0 is satisfied. It can also be shown that whether the drift is outward or inward depends on the choice of initial phase φ_*mn*0_ between the island and particles. There is a similar picture for parallel acceleration. The behaviors of *v*_∥_, $v_{r}^{\tilde{B}}$ and $\dot{v}_{\parallel }^{\mathrm{mirror},\tilde{B}}$ are shown in Fig. 10. The above loss mechanism has been invoked to explain the results of an experiment in ASDEX-U [40], where it was shown that the loss of EPs due to NTMs is caused by resonance for ICRF-heated plasmas, while it is caused by drift island overlap for NBI-heated plasmas. In ASDEX-U, losses of EIs due to TMs were measured to be as high as 30 MW m^−2^ and were attributed mainly to resonance [43]. Similar experimental results were found in DIII-D and have also been modeled [12,41].

EP losses in the presence of MIs have been found to be significant [12,36,38,40], and depend on the location and width of these islands. In the experiments that have shown dramatic EP losses, the MI locations are such that *r_s_*/*a* > 0.5 and their width is large. For example, in experiments in DIII-D, the profiles of EIs, like the density profile, were significantly different in the presence of an MI compared with those in its absence, with the amplitude of the (2,1) TM being more than 10% of the minor radius. This poor confinement of EIs may not arise in the case of magnetic fusion experiments, where the minor radius is large and so the ratio of the amplitude of TMs to the minor radius will be much smaller, as a consequence of which the transport of EIs will be reduced. However, even though the losses will be reduced, the energy and momentum of EPs will be redistributed in the presence of an MI such that they become less than the threshold for overlap of drift islands [41]. The profiles of density, driven torque and driven current will therefore be changed, which will affect plasma performance.

We have described above the transport mechanisms of circulating and trapped EPs due to TMs. It had been thought that resonance between EPs and TMs rarely occurs owing to the low frequency of these modes. However, rapid frequency chirping during TMs or NTMs has been observed in TFTR [27], ASDEX-U [28], DIII-D [31], EAST [29] and HL-2A [30]. During the chirping process, some EPs are expelled. In TFTR, measurable reductions of up to 1% in the neutron rate have been found [27]. Similar observations have been made in DIII-D [31]. The frequency chirping indicates that a strong resonance between EPs and TMs occurs, i.e. EIs do resonate with TMs in these experiments. The transport mechanism may then be similar to resonant loss due to the mode frequency beating with the EP bounce/transit or precessional frequency. A typical example is fishbone-induced loss, where the mode frequency matches the precessional frequency for low-frequency fishbones. In this case, the radial $\mathbf {E} \times \mathbf {B}$ drift velocity associated with the mode frequency becomes important. This will give rise to drift resonant TEPs, which will always be directed in the same direction during the particle bounce periods because of phase locking with the mode. The resonant particles will thus be expelled.

## CONCLUSIONS, DISCUSSION AND OUTLOOK

We have reviewed the interaction between EPs and TMs mainly from two perspectives: (i) the influence of EPs on TMs and (ii) the transport and confinement of EPs by TMs.

To consider the influence of EPs on TMs, we have used a hybrid model in which the modes and particles are described using MHD and kinetic theory, respectively. The physics of EPs is included in the MHD model via the pressure tensor term in the momentum equation and the driven current in Ohm’s law. Based on boundary layer theory, the TMs are analyzed by separating the region of interest into an outer (ideal) region and an island region (or resistive layer). The effects of EPs on TMs are then analyzed separately in these two regions. Basically, the main effect of EPs is through the perturbed current, since TMs are driven by the plasma current gradient. In this review, we have considered the limit in which the island width is much smaller than the orbital width, which is always the case for the linear phase, the early nonlinear phase and the onset threshold of NTMs. The orbits of EPs are then almost completely confined to the outer region where their direct interaction with the TM takes place. An instability criterion for the TM in which the effects of EPs are taken into account has been derived and analyzed, considering the effects of both circulating and trapped EPs. These effects have also been simulated by a hybrid MHD code, and the simulation results confirm the analytical results. In the island region, the EPs have an indirect effect through the quasineutrality condition. The response of EPs in the island region can be neglected owing to orbital averaging, since their orbits are almost completely in the outer region. In addition to the effect of EPs, the effect of toroidal rotation on TMs should be considered, since this rotation is naturally driven by NBI. Some discrepancies in the experimental results can be explained qualitatively by a combination of the effects of EPs and toroidal rotation. Based on the analysis presented here, the mechanisms underlying the behavior of EPs has been explored. Furthermore, a method for the control of NTMs has been proposed.

There are some issues that need to be pointed out here.

1. In previous studies, it has been assumed that the width of the MI is much smaller than the orbital width. However, in the later nonlinear period or on saturation of NTMs, the size of the MI will increase and become comparable to or larger than the orbital width. In this case, the physics will be different. In particular, if the MI is comparable to the orbital width, the orbits will cross the island region and the outer region, and the physics will become very complex. If the MI is larger than the orbital width then behavior of EPs may be similar to that of thermal particles. To investigate this, a simulation using the GTC with a perturbed parallel current of EIs added to Ampére’s law has been performed [23], and the results have been compared with those from an experiment in DIII-D [12]. However, there are no simulation codes for NTMs that self-consistently include both the EP physics and toroidal rotation. Up to now, most codes simulating the effects of EPs on plasma instabilities have not considered the kinetic effects of EPs on equilibrium, although these effects are important for TMs. It is therefore an urgent task to develop a self-consistent code that takes these effects into account.
2. Frequency chirping during TMs or NTMs has been found in many tokamaks. This indicates strong resonance between EPs and TM. Although some work has been devoted to this phenomenon, the explicit mechanism is still unclear, and further studies are needed.
3. Another important issue is the interaction between TMs and modes driven or excited by EPs. These particles can drive or excite other MHD instabilities, which will interact with TMs. For example, in the case of interaction between TMs and Alfvén eigenmodes (AEs), the formation of MI will change the continuum spectrum [77]. Pairs of beta-induced Alfvén eigenmodes (BAEs) in the presence of TMs without EPs are frequently observed in some tokamaks [30,78–80], and some studies have been devoted to explaining this phenomenon [32,81]. BAEs, often driven by EPs, are an important issue in MCF plasmas. An MI will change the profile of EPs, steepening the gradient near the island separatrix. As a consequence, BAEs will be more easily excited in the presence of an island, and they will in turn affect the transport of EIs. The interaction of other AEs, such as toroidal AEs, with TMs has been studied in [82,83].

We have reviewed the transport of both circulating and trapped EPs by TMs. In the case of CEPs, the drift islands formed in phase space owing to magnetic drift can overlap if the MI width and the particle energy are sufficiently large. This overlap of drift islands leads to orbital stochasticity, and this then causes the expulsion of EPs. For TEPs, magnetic perturbation plays a similar role to a ripple field in inducing losses of EPs. In addition, a special resonance resulting from beating of the bounce and precessional frequencies can match the mode frequency and induce loss of EPs. In general, the loss of EPs depends on the location and amplitude of the MI. If the island is close to the boundary and the amplitude is large, the loss of EPs induced by the island is dramatic. For smaller islands, although the loss is reduced, there are still substantial changes to the profiles of EPs. This is predicted to occur in large tokamaks like ITER, where the minor radius is large and so the ratio of the MI width to minor radius is small. Losses of EPs need to be quantified by simulations. Codes are now available for such simulations, including ORBIT [76], OFMIC [84] and GOURDON [85] for example. In these codes, equilibrium states and magnetic perturbation are used as inputs, and the perturbation is kept constant. This is valid for nonresonant cases. However, when there is resonance between EPs and the TM, the particles may have a significant effect on the mode, and these codes do not produce satisfactory results. To deal with this issue, the TRANSP-‘Kick’ code has been developed, in which the distribution of EPs due to magnetic perturbation is allowed to evolve, and this code has been shown to be valid in the presence of resonance [41]. Models that take account of mode evolution are also being developed, such as the RBQ1D code [86], which describes EP transport induced by Alfvén eigenmodes. It should be noted that Alfvén eigenmodes can become complex under the combined effects of MIs and other causes of particle loss, such as ripple fields. As pointed out above, TMs can interact strongly with modes that are driven or excited by EPs, and this can affect the transport of EPs, as has been observed in some experiments [87,88].

Another issue is the physics of runaway electrons. Weak confinement is desirable to minimize the number of runaway electrons and to avoid their localized and concentrated impact on the first wall. During disruptions such as thermal and current quenches, excitation of resistive instabilities is inevitable, owing to peaking of the current profile and growth of resistivity. In this process, there is strong interaction between TMs and runaway electrons. For example, resonant magnetic perturbation is often used to produce MIs to affect the confinement of runaway electrons. However, in this review, we have not discussed runaway electron physics.

## References

[1] Furth HP , Killeen J, Rosenbluth MN. Finite resistivity instabilities of a sheet pinch. Phys Fluids 1963; 6: 459–84. 10.1063/1.1706761

[2] Chang Z , Callen JD, Fredrickson ED et al. Observation of nonlinear neoclassical pressure-gradient-driven tearing modes in TFTR. Phys Rev Lett 1995; 74: 4663–6. 10.1103/PhysRevLett.74.4663 10058567

[3] Hender TC , Wesley JC, Bialek J et al. Chapter 3: MHD stability, operational limits and disruptions. Nucl Fusion 2007; 47: s128–202. 10.1088/0029-5515/47/6/S03

[4] Chen L , Zonca F. Physics of Alfvén waves and energetic particles in burning plasmas. Rev Mod Phys 2016; 88: 015008. 10.1103/RevModPhys.88.015008

[5] Todo Y . Introduction to the interaction between energetic particles and Alfven eigenmodes in toroidal plasmas. Rev Mod Plasma Phys 2019; 3: 1. 10.1007/s41614-018-0022-9

[6] Lu ZX , Wang X, Lauber P et al. Mode structure symmetry breaking of energetic particle driven beta-induced Alfvén eigenmode. Phys Plasmas 2018; 25: 012512. 10.1063/1.5006678

[7] Fasoli A , Gormenzano C, Berk HL et al. Chapter 5: physics of energetic ions. Nucl Fusion 2007; 47: s264–84. 10.1088/0029-5515/47/6/S05

[8] Buttery RJ , Haye RJ, Gohil P et al. The influence of rotation on the β(*N*) threshold for the 2/1 neoclassical tearing mode in DIII-D. Phys Plasmas 2008; 15: 056115. 10.1063/1.2894215

[9] Fietz S , Maraschek M, Zohm H et al. Influence of rotation on the (*m, n*) = (3, 2) neoclassical tearing mode threshold in the ASDEX Upgrade. Plasma Phys Control Fusion 2013; 55: 085010. 10.1088/0741-3335/55/8/085010

[10] Gerhardt SP , Brennan DP, Buttery R et al. Relationship between onset thresholds, trigger types and rotation shear for the *m*/*n* = 2/1 neoclassical tearing mode in a high-β spherical torus. Nucl Fusion 2009; 49: 032003. 10.1088/0029-5515/49/3/032003

[11] Anderson JK , Almagri AF, Hartog DJ et al. Fast ion confinement and stability in a neutral beam injected reversed field pinch. Phys Plasmas 2013; 20: 056102. 10.1063/1.4801749

[12] Heidbrink WM , Bardoczi L, Collins CS et al. The phase-space dependence of fast-ion interaction with tearing modes. Nucl Fusion 2018; 58: 082027. 10.1088/1741-4326/aab7b6

[13] Hegna CC , Bhattacharjee A. Suppression of magnetic islands by energetic ions in toroidal plasmas. Phys Fluid B 1990; 2: 1804–14. 10.1063/1.859452

[14] Takahashi R , Brennan DP, Kim CC. Kinetic effects of energetic particles on resistive MHD stability. Phys Rev Lett 2009; 102: 135001. 10.1103/PhysRevLett.102.135001 19392362

[15] Cai HS , Wang SJ, Xu YF et al. Influence of energetic ions on tearing modes. Phys Rev Lett 2011; 106: 075002. 10.1103/PhysRevLett.106.075002 21405521

[16] Cai HS , Fu GY. Hybrid simulation of energetic particle effects on tearing modes in tokamak plasmas. Phys Plasmas 2012; 19: 072506. 10.1063/1.4736956

[17] Cai HS , Lin L, Ding WX et al. Influence of energetic ions on tearing modes in a reversed field pinch. Plasma Phys Control Fusion 2015; 57: 025021. 10.1088/0741-3335/57/2/025021

[18] Halfmoon MR , Brennan DP. A model of energetic ion effects on pressure driven tearing modes in tokamaks. Phys Plasmas 2017; 24: 062501. 10.1063/1.4984772

[19] Zhang XX , Cai HS, Wang ZX. Influence of deeply trapped energetic ions on tearing modes. Phys Plasmas 2019; 26: 062505. 10.1063/1.5058733

[20] Cai HS . Influence of energetic ions on neoclassical tearing modes. Nucl Fusion 2016; 56: 126016. 10.1088/0029-5515/56/12/126016

[21] Cai HS , Cao JT, Li D. Influence of toroidal rotation on tearing modes. Nucl Fusion 2017; 57: 056006. 10.1088/1741-4326/aa5fd3

[22] Cai HS , Cao JT. Influence of toroidal rotation on magnetic islands in tokamaks. Nucl Fusion 2018; 58: 036008. 10.1088/1741-4326/aaa55e

[23] Tang X , Lin Z, Heidbrink WW et al. Gyrokinetic particle simulations of interactions between energetic particles and magnetic islands induced by neoclassical tearing modes. Phys Plasmas 2020; 27: 032508. 10.1063/1.5126681

[24] Pinches SD , Chapman IT, Lauber PW et al. Energetic ions in ITER plasmas. Phys Plasmas 2015; 22: 021807. 10.1063/1.4908551

[25] Wang XQ . Influence of circulating fast ions on nonlinear kink-tearing modes in tokamak plasmas. Europhys Lett 2016; 115: 45003. 10.1209/0295-5075/115/45003

[26] Wang XQ , Wang XG. Effects of energetic ions on double tearing modes in reversed shear plasmas. Nucl Fusion 2017; 57: 016039. 10.1088/1741-4326/57/1/016039

[27] Fredrickson ED . Observation of spontaneous neoclassical tearing modes. Phys Plasmas 2002; 9: 548–59. 10.1063/1.1435003

[28] Sesnic S , Günter S, Gude A et al. Interaction of fast particles with neoclassical tearing modes. Phys Plasmas 2000; 7: 935–9. 10.1063/1.873891

[29] Li E , Igochine V, Xu L et al. The dynamics of a neoclassical tearing mode (NTM) influenced by energetic ions on EAST. Plasma Phys Control Fusion 2016; 58: 045012. 10.1088/0741-3335/58/4/045012

[30] Chen W , Zhu XL, Wang F et al. Resonant interaction of tearing modes with energetic-ions resulting in fishbone activities on HL-2A. Nucl Fusion 2019; 59: 096037. 10.1088/1741-4326/ab2bc6

[31] Liu DY , Heidbrink WW, Podestà M et al. Cause and impact of low-frequency chirping modes in DIII-D hybrid discharges. Nucl Fusion 2020; 60: 112009. 10.1088/1741-4326/ab868c

[32] Marchenko VS , Lutsenko VV. Interaction of neoclassical tearing modes with trapped fast ions. Phys Plasmas 2001; 8: 4834–8. 10.1063/1.1407283

[33] Cai HS . Frequency chirping of neoclassical tearing modes by energetic ions. Nucl Fusion 2021; 61: 126012. 10.1088/1741-4326/ac2b77

[34] Zhu XL , Chen W, Wang F et al. Hybrid-kinetic simulation of resonant interaction between energetic-ions and tearing modes in a tokamak plasma. Nucl Fusion 2020; 60: 046023. 10.1088/1741-4326/ab742f

[35] Zhang XX , Gao BF, Cai HS et al. Excitation of 2/1 fishbone-like modes by trapped energetic ions. Plasma Phys Control Fusion 2020; 62: 085010. 10.1088/1361-6587/ab9a11

[36] Zweben SJ , Duvall RE, Fredrickson ED et al. MeV ion confinement in the TFTR tokamak. Phys Fluids B 1990; 2: 1411–4. 10.1063/1.859565

[37] Mynick HE . Transport of energetic ions by low-n magnetic perturbations. Phys Fluids B 1993; 5: 1471–81. 10.1063/1.860886

[38] Forest CB , Ferron JR, Gianakon T et al. Reduction in neutral beam driven current in a tokamak by tearing modes. Phys Rev Lett 1997; 79: 427–30. 10.1103/PhysRevLett.79.427

[39] Medley SS , Gorelenkov N, Andre R et al. MHD-induced energetic ion loss during H-mode discharges in the national spherical torus experiment. Nucl Fusion 2004; 44: 1158–75. 10.1088/0029-5515/44/11/002

[40] García-Muñnoz M , Martin P, Fahrbach H et al. NTM induced fast ion losses in ASDEX Upgrade. Nucl Fusion 2007; 47: L10–5. 10.1088/0029-5515/47/7/L03

[41] Bardóczi L , Podestà M, Heidbrink WW et al. Quantitative modeling of neoclassical tearing mode driven fast ion transport in integrated TRANSP simulations. Plasma Phys Control Fusion 2019; 61: 055012. 10.1088/1361-6587/ab0f08

[42] Heidbrink WW , White RB. Mechanisms of energetic-particle transport in magnetically confined plasmas. Phys Plasmas 2020; 27: 030901. 10.1063/1.5136237

[43] Galdon-Quiroga J , García-Muñnoz M, Sanchis-Sanchez L et al. Velocity space resolved absolute measurement of fast ion losses induced by a tearing mode in the ASDEX Upgrade tokama. Nucl Fusion 2018; 58: 036005. 10.1088/1741-4326/aaa33b

[44] Poli E , García-Muñoz M, Fahrbach H et al. Observation and modeling of fast trapped ion losses due to neoclassical tearing modes. Phys Plasmas 2008; 15: 032501. 10.1063/1.2890771

[45] Bonofiglo PJ , Anderson JK, Gobbin M et al. Fast ion transport in the quasi-single helical reversed-field pinch. Phys Plasmas 2019; 26: 022502. 10.1063/1.5084059 31491308

[46] Hao BL , White R, Gao X et al. Numerical investigation of alpha particle confinement under the perturbation of neoclassical tearing modes and toroidal field ripple in CFETR. Nucl Fusion 2021; 61: 046035. 10.1088/1741-4326/abe4e8

[47] Hao BL , Chen W, Li GQ et al. Numerical simulation of synergistic effect of neoclassical tearing mode and toroidal field ripple on alpha particle loss in China Fusion Engineering Testing Reactor. Acta Phys Sin 2021; 70: 115201. 10.7498/aps.70.20201972

[48] Yu LM , Xue EB, Zhang DB et al. Simulation of the loss of passing fast ions induced by magnetic islands in EAST tokamak plasmas. AIP Adv 2021; 11: 025020. 10.1063/5.0032049

[49] Yang J , Podestà M, Fredrickson ED. Synergy of coupled kink and tearing modes in fast ion transport. Plasma Phys Control Fusion 2021; 63: 045003. 10.1088/1361-6587/abd9e4

[50] Khan M , Schoepf K, Goloborodko V et al. Symplectic simulations of radial diffusion of fast alpha particles in the presence of low-frequency modes in rippled tokamaks. J Fusion Energy 2017; 36: 40–7. 10.1007/s10894-016-0120-z

[51] Waddel BV , Carreras B, Hicks HR et al. Nonlinear interaction of tearing modes in highly resistive tokamak. Phys Fluids 1979; 22: 896–910. 10.1063/1.862685

[52] Li D , Yang W, Cai HS. On theoretical research for nonlinear tearing mode. Plasma Sci Technol 2018; 20: 094002. 10.1088/2058-6272/aabde4

[53] Feng HY , Zhang WL, Dong C et al. Verification of linear resistive tearing instability with gyrokinetic particle code VirtEx. Phys Plasmas 2017; 24: 102125. 10.1063/1.4999166

[54] Liu DJ , Bao J, Han T et al. Verification of gyrokinetic particle simulation of current-driven instability in fusion plasmas. III. Collisionless tearing mode. Phys Plasmas 2016; 23: 022502. 10.1063/1.4941094

[55] Rutherford PH . Nonlinear growth of the tearing mode. Phys Fluids 1973; 16: 1903–8. 10.1063/1.1694232

[56] Cai HS , Li D, Cao JT. Influence of ion orbit width on onset threshold of neoclassical tearing modes. Phys Plasmas 2015; 22: 102512. 10.1063/1.4934214

[57] Dong G , Lin Z. Effects of magnetic islands on bootstrap current in toroidal plasmas. Nucl Fusion 2017; 57: 036009. 10.1088/1741-4326/57/3/036009

[58] Imada K , Wilson HR, Connor JW et al. Nonlinear kinetic ion response to small scale magnetic islands in tokamak plasmas: neoclassical tearing mode threshold physics. Phys Rev Lett 2018; 121: 175001. 10.1103/PhysRevLett.121.175001 30411924

[59] Maraschek M , Gantenbein G, Yu Q et al. Enhancement of the stabilization efficiency of a neoclassical magnetic island by modulated electron cyclotron current drive in the ASDEX Upgrade tokamak. Phys Rev Lett 2007; 98: 025005. 10.1103/PhysRevLett.98.025005 17358617

[60] Li JC , Xiao CJ, Lin ZH et al. Effects of electron cyclotron current drive on magnetic islands in tokamak plasmas. Phys Plasmas 2017; 24: 082508. 10.1063/1.4996021

[61] Wang XJ , Yu QQ, Zhang XD et al. Numerical modelling on stabilizing large magnetic island by RF current for disruption avoidance. Nucl Fusion 2018; 58: 016045. 10.1088/1741-4326/aa944e

[62] Wilson HR , Connor JW, Hastie RJ et al. Threshold for neoclassical magnetic islands in a low collision frequency tokamak. Phys Plasmas 1996; 3: 248–65. 10.1063/1.871830

[63] Fischer R , Giannone L, Lackner K et al. Effect of measured toroidal flows on tokamak equilibria. In: 42nd EPS Conference on Plasma Physics, Lisbon, Portugal. European Physical Society, 2015, 257–60.

[64] Sen A , Chandra D, Kaw P. Tearing mode stability in a toroidally flowing plasma. Nucl Fusion 2013; 53: 053006. 10.1088/0029-5515/53/5/053006

[65] Chandra D , Thyagaraja A, Sen A et al. Modelling and analytic studies of sheared flow effects on tearing modes. Nucl Fusion 2015; 55: 053016. 10.1088/0029-5515/55/5/053016

[66] Wang S , Ma ZW. Influence of toroidal rotation on resistive tearing modes in tokamaks. Phys Plasmas 2015; 22: 122504. 10.1063/1.4936977

[67] Ren ZH , Liu JY, Wang F et al. Influence of toroidal rotation on the tearing mode in tokamak plasmas. Plasma Sci Technol 2020; 22: 065102. 10.1088/2058-6272/ab77d4

[68] Shao JR , Liu H, Xu YH et al. Effect of the toroidal flow and flow shear on the *m*/*n* = 2/1 tearing mode in J-TEXT tokamak. Plasma Phys Control Fusion 2021; 63: 065017. 10.1088/1361-6587/abf85e

[69] Liu T , Wei L, Wang F et al. Coriolis force effect on suppression of neo-classical tearing mode triggered explosive burst in reversed magnetic shear tokamak plasmas. Chin Phys Lett 2021; 38: 045204. 10.1088/0256-307X/38/4/045204

[70] Sakamoto Y , Fujita T, Ide S et al. Stationary high confinement plasmas with large bootstrap current fraction in JT-60U. Nucl Fusion 2005; 45: 574–80. 10.1088/0029-5515/45/7/004

[71] Grave JP . Toroidal drift precession and wave–particle interaction in shaped tokamaks with finite beta and neoclassical equilibrium effects. Plasma Phys Control Fusion 2013; 55: 074009. 10.1088/0741-3335/55/7/074009

[72] Qu ZS , Hole MJ, Fitzgerald M. Modeling the effect of anisotropic pressure on tokamak plasmas normal modes and continuum using fluid approaches. Plasma Phys Control Fusion 2015; 57: 095005. 10.1088/0741-3335/57/9/095005

[73] Gao BF , Cai HS, Wang F et al. Influence of trapped energetic ions on low-frequency magnetohydrodynamic instabilities with reversed shear profile. Phys Plasmas 2021; 28: 012104. 10.1063/5.0034690

[74] Berk HL , Breizman BN, Petviashvili NV. Spontaneous hole-clump pair creation in weakly unstable plasmas. Phys Lett A 1997; 234: 213–8. 10.1016/S0375-9601(97)00523-9

[75] Hezaveh H , Qu ZS, Breizman BN et al. Long range frequency chirping of Alfven eigenmodes. Nucl Fusion 2020; 60: 056014. 10.1088/1741-4326/ab7d18

[76] White R , Chance M. Hamiltonian guiding center drift orbit calculation for plasmas of arbitrary cross section. Phys Fluids 1984; 27: 2455–67. 10.1063/1.864527

[77] Biancalani A , Chen L, Pegoraro F et al. Continuous spectrum of shear Alfvén waves within magnetic islands. Phys Rev Lett 2010; 105: 095002. 10.1103/PhysRevLett.105.095002 20868168

[78] Buratti P , Smeulders P, Zonca F et al. Observation of high-frequency waves during strong tearing mode activity in FTU plasmas without fast ions. Nucl Fusion 2005; 45: 1446–50. 10.1088/0029-5515/45/11/027

[79] Liu L , Zhuang G, Hu QM et al. Beta-induced Alfvén eigenmodes destabilized by resonant magnetic perturbations in the J-TEXT tokamak. Nucl Fusion 2019; 59: 126022. 10.1088/1741-4326/ab4090

[80] Xu M , Kong DF, Liu AD et al. Experimental observation of the localized coupling between geodesic acoustic mode and magnetic islands in tokamak plasmas. Nucl Fusion 2021; 61: 036034. 10.1088/1741-4326/abd72d

[81] Cai HS , Gao BF, Xu M. Excitation of beta-induced Alfvén eigenmodes by the coupling between geodesic acoustic mode and magnetic island. Nucl Fusion 2021; 61: 036029.

[82] Zhu J , Ma ZW, Wang S et al. Nonlinear dynamics of toroidal Alfvén eigenmodes in the presence of tearing modes. Nucl Fusion 2018; 58: 046019. 10.1088/1741-4326/aaae7e

[83] Shi PW , Qiu ZY, Chen W et al. Nonlinear mode coupling induced high frequency axisymmetric mode on the HL-2A tokamak. Nucl Fusion 2019; 59: 086001. 10.1088/1741-4326/ab1af8

[84] Shinohara K , Kawashima H, Tsuzuki K et al. Effects of complex magnetic ripple on fast ions in JFT-2M ferritic insert experiments. Nucl Fusion 2003; 43: 586–93. 10.1088/0029-5515/43/7/312

[85] Gourdon C . Programme Optimise de Calculs Numeriques Dans le Configurations Magnetique Toroidales. Centre d’Etudes Nucléaires de Fontenay aux Roses, 1970.

[86] Gorelenkov N , Duarte V, Podesta M et al. Resonance broadened quasi-linear (RBQ) model for fast ion distribution relaxation due to Alfvénic eigenmodes. Nucl Fusion 2017; 58: 082016. 10.1088/1741-4326/aac72b

[87] Shi PW , Chen W, Shi ZB et al. Destabilization of toroidal Alfvén eigenmode during neutral beam injection heating on HL-2A. Phys Plasma 2017; 24: 042509.

[88] Madsen B , Salewski M, Heidbrink WW et al. Tomography of the positive-pitch fast-ion velocity distribution in DIII-D plasmas with Alfven eigenmodes and neoclassical tearing modes. Nucl Fusion 2020; 60: 066024. 10.1088/1741-4326/ab82b5Au

